# Uncovering the Protein Lysine and Arginine Methylation Network in Arabidopsis Chloroplasts

**DOI:** 10.1371/journal.pone.0095512

**Published:** 2014-04-18

**Authors:** Claude Alban, Marianne Tardif, Morgane Mininno, Sabine Brugière, Annabelle Gilgen, Sheng Ma, Meryl Mazzoleni, Océane Gigarel, Jacqueline Martin-Laffon, Myriam Ferro, Stéphane Ravanel

**Affiliations:** 1 Université Grenoble Alpes, Laboratoire de Physiologie Cellulaire & Végétale, Grenoble, France; 2 CNRS, UMR5168, Grenoble, France; 3 CEA, iRTSV, Laboratoire de Physiologie Cellulaire & Végétale, Grenoble, France; 4 INRA, USC1359, Grenoble, France; 5 Université Grenoble Alpes, Grenoble, France; 6 CEA, iRTSV, Biologie à Grande Echelle, Grenoble, France; 7 INSERM, U1038, Grenoble, France; Universität Stuttgart, Germany

## Abstract

Post-translational modification of proteins by the addition of methyl groups to the side chains of Lys and Arg residues is proposed to play important roles in many cellular processes. In plants, identification of non-histone methylproteins at a cellular or subcellular scale is still missing. To gain insights into the extent of this modification in chloroplasts we used a bioinformatics approach to identify protein methyltransferases targeted to plastids and set up a workflow to specifically identify Lys and Arg methylated proteins from proteomic data used to produce the Arabidopsis chloroplast proteome. With this approach we could identify 31 high-confidence Lys and Arg methylation sites from 23 chloroplastic proteins, of which only two were previously known to be methylated. These methylproteins are split between the stroma, thylakoids and envelope sub-compartments. They belong to essential metabolic processes, including photosynthesis, and to the chloroplast biogenesis and maintenance machinery (translation, protein import, division). Also, the *in silico* identification of nine protein methyltransferases that are known or predicted to be targeted to plastids provided a foundation to build the enzymes/substrates relationships that govern methylation in chloroplasts. Thereby, using *in vitro* methylation assays with chloroplast stroma as a source of methyltransferases we confirmed the methylation sites of two targets, plastid ribosomal protein L11 and the β-subunit of ATP synthase. Furthermore, a biochemical screening of recombinant chloroplastic protein Lys methyltransferases allowed us to identify the enzymes involved in the modification of these substrates. The present study provides a useful resource to build the methyltransferases/methylproteins network and to elucidate the role of protein methylation in chloroplast biology.

## Introduction

Protein methylation has emerged as an important and widespread post-translational modification affecting almost all basic cellular processes in prokaryotes and eukaryotes. It provides important functional diversity and regulatory complexity. Indeed, methylation can affect the side chain of several residues as well as the amino and carboxyl termini of proteins. In eukaryotes, methylation is predominantly found on lysine (Lys) and arginine (Arg) residues [1]. Lys and Arg can be multiply methylated (from one to three methyl groups in case of Lys and one to two methyl groups in case of Arg) and the different levels of methylation correlate with distinct effects. Also, various sites of methylation within a target protein can have opposite biological functions and can compete or cross-talk with other modifications (e.g. acetylation or ubiquitination) [2].

Methylation of the Lys ε-amino group is catalyzed by protein Lys methyltransferases (PKMTs). The majority of PKMTs possess a conserved and well-defined catalytic domain named SET [3], [4]. Recent studies have identified a new group of distantly related PKMTs belonging to the superfamily of seven-beta-strand methyl-transferases [1], [5]. Each PKMT is often associated with a limited number of targets and can generate mono-, di- or tri-methylated lysyl residues (designated K_me1_, K_me2_, and K_me3_, respectively). Protein Arg methyltransferases (PRMTs) have a seven-beta-strand structural fold and catalyze the transfer of one or two methyl groups to the distal nitrogen atoms of the guanidino group of Arg residues, resulting in either monomethyl- or dimethyl-Arg (R_me1_, R_me2_) [6]. The substrate specificity of PRMTs is often broader than PKMTs. Both types of methyltransferases utilize *S*-adenosylmethionine (AdoMet) as methyl donor and release *S*-adenosylhomocysteine during catalysis. Lys and Arg methylations were regarded as enzymatically irreversible until the discovery of two classes of Lys demethylases [7]. The presence of Arg demethylases is still a matter of debate [8]. Thus, protein Lys and Arg methylation can be either a dynamic process and serve regulatory purposes or a static modification that extends the functional repertoire of amino acids.

By far, most of our understanding of protein Lys and Arg methylation comes from studies of histones and their role in epigenetics [9]. During the last years, the biochemical characterization of purified methyltransferases, for example using peptide arrays [10], together with the development of antibodies specific to free methylated Lys/Arg and the advances in mass spectrometry (MS)-based approaches led to the identification of dozens of non-histone methylated proteins in yeast, animal and trypanosome cells [11]–[16]. These proteins are involved in diverse cellular processes including transcriptional regulation, RNA processing, translation, intracellular protein trafficking, cellular signaling or metabolism. In most cases, the biological significance of non-histone proteins methylation is poorly understood. However, there are several non-histone substrates, e.g. the transcription factor p53 [17] or the DNA-modifying enzyme DNMT1 [18], for which the modification was shown to have important impact on protein function. These functional studies indicated that methylation plays key roles in the regulation of protein-protein interactions, protein-nucleic acids interactions or protein stability.

Thanks to advances in proteomic technologies and improved plant genomic resources, plastids and particularly chloroplasts are among the best characterized cell organelles at the proteome level [19]. The plastid proteome atlas is improved continuously to provide biologically useful information including sub-plastidial localization, cellular specialization, steady-state protein abundance or post-translational modifications. For example, several reversible modifications including phosphorylation, acetylation of internal Lys residues, and redox-dependent modifications have been established as key regulators of plastid metabolism, signaling and gene expression [20]–[24]. To date, little is known about the extent of protein methylation in chloroplasts. Methylation of a number of polypeptides located in the stroma and thylakoids from pea and spinach chloroplasts was reported almost 25 years ago [25], [26]. Only a few of these proteins have been yet identified and their methylation sites determined. In some plant species (e.g. pea and tobacco) the main stromal methylprotein is the large subunit of Rubisco (RbcL), the enzyme responsible for CO_2_ fixation in the Calvin cycle [27], [28]. Trimethylation of RbcL at Lys14 is catalyzed by the large subunit Rubisco methyltransferase (LSMT), a highly conserved SET-domain containing PKMT found in all plant species [29]. In *Arabidopsis thaliana*, RbcL is not methylated at Lys14 and the physiological substrates of the LSMT-like enzyme are chloroplastic isoforms of fructose 1,6-bisphosphate aldolases [30]. Plastid ribosomal protein L11 (PRPL11) from spinach [31] and ferredoxin-NADP reductase of the unicellular alga *Chlamydomonas reinhardtii* [32] were also shown to be trimethylated at Lys residues. Last, it should be mentioned that the small subunit of Rubisco from various plant species [33] and the plastid ribosomal proteins L2 and L16 from spinach [31], [34] were found methylated on the α-amino group of their N-terminal residues. With the exception of RbcL and aldolases, the enzymes responsible for these modifications have not been yet identified. Also, the role of methylation is still not known for all chloroplastic methylproteins. Despite limited knowledge, the physiological significance of protein methylation in chloroplasts is illustrated by the lack of chloroplast differentiation and albino phenotype of a mutant impaired in the plastid-located SET-domain methyltransferase PTAC14 [35].

In this study, we aimed to get insights into the extent of protein methylation in the biology of chloroplasts. For this purpose, we first used a bioinformatics approach to identify the set of protein Lys and Arg methyltransferases targeted to plastids. Second, we set up a workflow to specifically identify Lys and Arg methylated proteins from proteomic data used to generate the Arabidopsis chloroplast proteome [36], [37]. Using this approach we identified 31 high-confidence Lys and Arg methylation sites from 23 chloroplastic proteins of diverse functional classes and sub-plastidial locations. The identified methylproteins belong to essential metabolic processes, including photosynthesis, and to the chloroplast biogenesis and maintenance machinery (translation, protein import, division). Using chloroplast stroma as a source of methyltransferases, we validated the methylation sites of two targets, PRPL11 and the β-subunit of ATP synthase (ATP-B). Also, a biochemical screening of recombinant chloroplastic protein Lys methyl-transferases allowed us to identify the enzymes involved in PRPL11 and ATP-B modification. Together, our results pave the way to build the methyltransferases/methylproteins relationships, which is predicted to be an important network in the regulation of chloroplast biogenesis and metabolism.

## Materials and Methods

### Materials

[methyl-^3^H]-AdoMet (70–85 Ci.mmol^−1^) was purchased from PerkinElmer Life Sciences. Ni Sepharose 6 Fast Flow was from GE Healthcare Bio-Sciences. Unless otherwise stated, other chemicals and reagents were from Sigma and of highest purity available. AdoMet was further purified by ion exchange chromatography on CM Sephadex C-25 [30].

### Purification and fractionation of chloroplasts

Chloroplasts from Arabidopsis (ecotype Columbia, Col-0), spinach and pea leaves were purified on Percoll gradients as previously described [30], [38]. Purity of chloroplast fractions was assessed by measurement of specific subcellular markers [39] and cross-contamination by mitochondrial and cytosolic proteins was found to be less than 10%. Intact chloroplasts were suspended into hypotonic medium and submitted to three freeze/thaw cycles to ensure complete lysis. Membranes (thylakoids plus envelope) were separated from soluble proteins (stroma) by centrifugation at 150,000×g for 30 min at 4°C through a 0.6 M sucrose cushion.

### Western blot analysis

Proteins from Arabidopsis, spinach and pea chloroplast subfractions were resolved by SDS-PAGE, electroblotted to nitrocellulose membranes, and probed with rabbit polyclonal antibodies to K_me3_ (ab76118, abcam), to K_me1/2_ (ab23366, abcam), or mouse polyclonal antibodies to R_me1/2_ (mab0002, Covalab). Immunoreactive proteins were visualized using the ECL Plus Western Blotting detection reagents and a Typhoon 9400 scanner (Amersham Biosciences).

### Identification of Lys and Arg methylation in the AT_CHLORO database

#### Database searching

The peak lists (494 runs) that constituted the AT_CHLORO database [36], [37] and data from additional samples (envelope, 3 biological replicates, and thylakoids, 2 biological replicates) were used. Thus, a total of 587 peak lists were searched against a target-decoy version of the complete Arabidopsis proteome (nuclear, mitochondrial, and plastid genome; TAIR v9.0; June 19, 2009; 33,518 entries) using the Mascot 2.3 search engine (Matrix Science). The target-decoy version of the database was generated by introducing a reversed version of the sequences. The search parameters were as in ref. [36] but with the introduction of methyl-specific aspects. In addition to the usual modifications (cysteine trioxidation, acetylation of protein N-termini, methionine oxidation and dioxidation) the set of variable modifications comprised the mono, di, or trimethylation of Lys and mono or dimethylation of Arg residues. The enzyme was set to Trypsin/P. As modified Lys and Arg might alter the efficiency of the protease, up to three miscleavages was allowed. The mass tolerances were set at 10 ppm for the precursor ions and at 0.8 Da for the fragment ions.

#### Parsing of results

Mascot search results were automatically filtered using the home-developed IRMa 1.25.0 software [40]. The following parameters were applied. (i) The number of report hits was fixed automatically to retrieve proteins with a *p*-value <0.05, as defined by Mascot. (ii) A cutoff score of 20 was applied for the peptides. (iii) Only peptides ranked first and with a homology threshold with *p*<0.05, as defined by Mascot, were kept. Every duplicated peptide sequences were conserved. Parsed results were then imported into a relational mass spectrometry identifications results database. Compiling the 587 different analyses in order to retrieve the list of peptides and non-redundant protein groups was performed using a home-made software.

#### Filtering of methylpeptides

**Figure 1:**
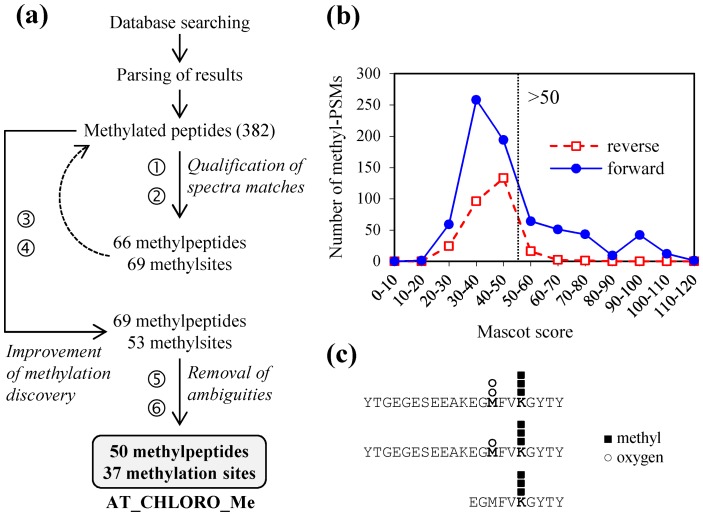
Filtering pipeline for the identification of methylpeptides in the AT_CHLORO datasets. (a) Following the initial database search and parsing of results, the workflow is composed of six steps combining automatic filtering and expert examination of spectra. 1/selection of methyl peptide-spectrum matches (PSMs) with score ≥ 50; 2/manual inspection to assess spectral quality; 3/selection of methyl-PSMs (score <50) with identical methylation sites; 4/removal of sites with only one PSM; 5/removal of ambiguities due to amino acid substitutions; 6/removal of ambiguities due to trimethylation vs. acetylation. The final AT_CHLORO_Me list consisted in methylpeptides validated from the mass spectrometry aspect. Peptide counts represent the number of distinct peptide sequences compiled from a larger number of PSMs. (b) Score distribution of methyl-PSMs matching a reverse or true (forward) Arabidopsis protein library. The threshold score of 50 in step 1 was selected from these distributions. (c) Illustration of the diversity among PSMs that identify a methylation site (step 3). In this example (FBA1 protein, At2g21330), three PSMs point out a trimethyl-Lys with two distinct overlapping peptide sequences and various modification patterns of a Met residue (mono or dioxidized).

In order to discriminate true methylated peptides from false positives, we applied the following filtering procedure (Fig. 1). Benefiting from searching a target-decoy version of the Arabidopsis database, a false discovery rate (FDR) for the methylpeptides was estimated according to the formula FDR = 2[n_rev_/(n_rev_+n_real_)], where n_rev_ is the number of methylated peptide-spectrum matches (PSMs) derived from reverse sequences and n_real_ the number of methyl-PSMs derived from real sequences [41]. To reduce this FDR, the first filtering step consisted in selecting PSMs above a Mascot score threshold of 50 (Fig. 1b). These PSMs were manually inspected to assess spectral quality, which in the end allowed us to delimitate a preliminary set of potentially methylated Lys/Arg sites. Next, we collected and manually checked the PSMs of score <50 that identified the same methylation sites. The rationale for this search is that different patterns of modifications (e.g. oxidation vs. dioxidation of methionine) or overlapping sequences generated by trypsin miscleavages can raise confidence in final methylation discovery (Fig. 1c) [11]. Additionally, collecting these spectra provided a spectral count (SC) value which could be taken as a rough estimation of the extent of methylation on each candidate site. Then, only methylation sites that were identified by at least two spectra were kept. Finally, methylation sites that relied solely on ambiguous PSMs due to sequence variants were discarded. Indeed, mono, di or trimethylation are isobaric to single amino acid substitutions and Mascot can assign PSMs to different sequences with almost similar scores. Also, ambiguity may arise from the mass shift of a trimethylation (42.04695) that is close to that of an acetylation (42.01056). In theory, setting the precursor mass tolerance at 10 ppm allowed by the Fourier-transform instrument precision is sufficient to discriminate between the two modifications [42], provided that the instrument was correctly calibrated. Thus, for each ambiguous trimethyl-peptide, we evaluated the error of its ion mass measurement relatively to the distribution of errors from all peptides with a score >40. We also checked MS/MS spectra for the presence of fragments corresponding to the neutral loss of trimethylamine (−59 Da), which is a signature of a trimethylation [42], [43].

### In silico prediction of protein subcellular localization and Lys/Arg methylation sites

The subcellular localization of Arabidopsis protein was analyzed using the SUBA3 search interface (http://suba.plantenergy.uwa.edu.au) [44], the AtSubP program (http://bioinfo3.noble.org/AtSubP/?dowhat=AtSubP) [45], and ChloroP (http://www.cbs.dtu.dk/services/ChloroP/) [46]. We used the following predictors for searching Lys and Arg methylation sites: MeMo v2.0 [47], BPB-PPMS (http://www.bioinfo.bio.cuhk.edu.hk/bpbppms/index.jsp) [48], MASA (http://masa.mbc. nctu. edu.tw/index.html) [49], PMeS [50], and PLMLA (http://bioinfo.ncu.edu.cn/inquiries _PLMLA. aspx) [51].

### Structural analysis of methylation sites accessibility

The 3D structures of proteins were modeled using Protein Homology/analogY Recognition Engine V2.0 *via* the Phyre^2^ server (www.sbg.bio.ic.ac.uk/phyre2/html/page.cgi?id=index) [52]. Visual inspection of 3D structures and methylation sites accessibility was conducted using PyMOL (DeLano Scientific, San Carlos, CA, USA).

### Production and purification of recombinant proteins

The full length cDNAs for PRPL11 (At1g32990) and GAPA1 (At3g26650) were obtained from the Arabidopsis Biological Resource Center (stocks U09645 and U21597, respectively) [53]. Sequences coding mature PRPL11 (starting at Ala63 to remove the chloroplast transit peptide) and mature GAPA1 (starting at Ala60) were amplified by PCR using the Phusion high fidelity DNA polymerase (Finnzymes) and primers containing the appropriate restriction sites (Table S1) for cloning into pET expression vectors. PRPL11 was cloned into pET20b(+) in frame with a C-terminal 6-His tag and GAPA1 into pET28b(+) in frame with a N-terminal 6-His tag. The sequence coding mature PrmA-like (At5g53920, starting at Ser63) was amplified using reverse-transcribed RNAs from Col-0 seedlings as templates and cloned into pET20b(+) in frame with a C-terminal 6-His tag. Sequences coding full-length ATP synthase β-subunit (ATP-B; AtCg00480) and mature PPKMT2 (At1g24610 starting at Ala23) were obtained by PCR amplification of total DNA and reverse-transcribed RNAs, respectively, prepared from Col-0 seedlings. Amplicons were cloned into pET30a(+) resulting in the production of recombinant ATP-B without tag and recombinant PPKMT2 with a C-terminal 6-His tag. The point mutation Lys447 to Ala in ATP-B was introduced using the QuickChange II site-directed mutagenesis kit (Stratagene) and suitable oligonucleotides (Table S1).

Recombinant proteins were produced in *Escherichia coli* Rosetta-2 cells (Stratagene) except for PRPL11 that was produced in the *E. coli* KNOK16 strain to avoid any possible methylation by the bacterial PrmA enzyme *in vivo* [54]. The *prma* knock-out strain was lysogenized with the helper phage (λDE3) harboring a copy of the T7 RNA polymerase (λDE3 lysogenization kit, Novagen) and co-transformed with the pRARE2 plasmid for efficient expression of recombinant PRPL11. Cells harboring the pET constructs were grown in Luria-Bertani medium at 37°C until mid-log phase and proteins production was induced by the addition of 0.4 mM isopropylthio-β-D-galactoside.

For ATP-B, cells were collected by centrifugation (4,000×*g*, 20 min) after 5 hours of growth at 37°C, suspended in buffer A (50 mM Tris-HCl, pH 8.0, 2 mM EDTA), and then disrupted by sonication. ATP-B was purified from inclusion bodies by extensive washes in buffer A, followed by solubilization in buffer A supplemented with 4 M urea and 2 mM dithiothreitol. Refolding was done by urea removal with three sequential steps of dialysis against buffered solutions containing decreasing amounts of urea and increasing amounts of glycerol, as described in ref. [55]. The ATP-B K_447_A mutant protein was produced and purified using the same procedure.

For recombinant FBA2, PRPL11, GAPA1, PrmA-like and PPKMT2, transformed cells were grown for 16 hours at 17°C after the addition of isopropylthio-β-D-galactoside. Harvested cells were suspended in buffer B (50 mM Tris-HCl, pH 8.0, 0.5 M NaCl, 10 mM imidazole and a cocktail of protease inhibitors), and disrupted by sonication. His-tagged proteins were purified from soluble protein extracts by chromatography onto Ni-Sepharose column according to the procedure described previously for the recombinant FBA2 protein [30].

### In vitro methylation assays

The incorporation of methyl groups from AdoMet into various protein substrates was determined essentially as described previously [30]. Assay mixtures contained phosphate buffer saline (pH 7.8), 20 µM [methyl-^3^H]-AdoMet, 100 nM *S*-adenosylhomocysteine hydrolase (Sigma-Aldrich), 80 µg chloroplast stroma from Arabidopsis Col-0 as a source of methyl-transferases, and various amounts of the protein substrates in a final volume of 30 µl. Assays were conducted at 30°C for 1 to 2 hours. Kinetic analyses with recombinant PPKMT2 or PrmA-like were done with 0.25–1 µg purified enzyme at 30°C. Reactions were terminated by the addition of 500 µl trichloroacetic acid 10% (w/v) and 5 µl sodium deoxycholate 1% (w/v). Radioactivity incorporated into proteins was determined by liquid scintillation and/or by phosphorimaging. For phosphorimage analyses, proteins were resolved by SDS-PAGE and transferred to ProBlott membranes (Applied Biosystems). Membranes were stained with Coomassie blue, dried, and exposed to a tritium storage phosphor screen (Molecular Dynamics) for 6 days before phosphorimage analysis using a Typhoon 9400 scanner (Amersham Biosciences).

### Mass spectrometry methods

Mass spectrometry analysis on recombinant proteins was carried out essentially as described in ref. [30] with minor modifications. For sample preparation, the oxidizing H_2_O_2_-treatment of gel bands was replaced by reduction with dithiothreitol for 45 min at 56°C and alkylation with iodoacetamide for 35 min at room temperature. LC-MS/MS raw data were acquired on a LTQ-Orbitrap (Velos) hybrid mass spectrometer (ThermoFisher) as in ref. [30]. Peak lists were generated with the Mascot Distiller version 2.4.3 software (Matrix Science) from the LC-MS/MS raw data. Using the Mascot 2.4 search engine (Matrix Science), we searched all MS/MS spectra against the target-decoy version of an updated compilation of the *A. thaliana* protein database provided by TAIR (nuclear, mitochondrial, and plastid genome; TAIR v10.0; December 14, 2010; 35,386 entries) and a home-made list of contaminants (260 entries). The set of allowed variable modifications were acetyl (N-termini), methionine oxidation and dioxidation, methyl (Lys, Arg), dimethyl (Lys, Arg), and trimethyl (Lys). In addition, carbamidomethyl cysteine was set as a fixed modification. Mascot search results were automatically filtered as described in ref. [30] with the IRMa 1.30.4 version software. Spectra of interest were checked manually to confirm sequence and modifications.

## Results and Discussion

### *In silico* identification of Arabidopsis protein Lys and Arg methyltransferases targeted to chloroplasts

More than 60 protein methyl-transferases have been previously annotated in the Arabidopsis genome, including nine PRMTs [8] and about 50 SET domain-containing PKMTs [4]. An increasing number of PKMTs with a seven-beta-strand structural fold is being identified in yeast and human cells [5], [56]–[59], but this subfamily is poorly documented in plants [60]. Using BLAST searches we identified ten Arabidopsis orthologs for these evolutionary conserved proteins. Then we analyzed the subcellular distribution of the full set of methyltransferases using the SUBA3 search interface [44] and the AtSubP program [45]. These predictions were combined with a review of proteomic data dedicated to Arabidopsis chloroplasts [36], [37], [61], [62].

We found that one out of nine PRMTs is likely targeted to plastids (Table 1). This protein (PRMT7, At4g16570) awaits biochemical and physiological characterization in plants [8]. In human, PRMT7 has been shown to methylate Arg residues on histones and non-histone protein substrates [63]. Six SET domain-containing PKMTs are known or predicted to be located into plastids (Table 1). Five of these proteins belong to subclass VII that is anticipated to methylate non-histone substrates [4]. The first enzyme, LSMT-like (At1g14030) catalyzes the methylation of fructose bisphosphate aldolases [30]. The second (PTAC14, At4g20130) belongs to the core complex of the plastid-encoded RNA polymerase [35]. It is essential for proper chloroplast biogenesis in early stages of development but its substrate is still unknown. The other proteins from subclass VII (At1g24610, At3g07670 and At5g14260) await functional characterization. Until their physiological substrates are identified we propose the nomenclature PPKMT1-3 for Plastid PKMTs, as in ref. [30]. The last SET-domain containing candidate (ATXR5, At5g09790) belongs to subclass IV and has a dual localization in plastids, with as yet unknown targets, and in the nucleus where it is able to methylate histone H3 [64], [65]. *In silico* analysis of putative Arabidopsis PKMTs with a seven-beta-strand structural fold suggested that two of them are targeted to plastids (Table 1). The first one is the ortholog of calmodulin Lys methyltransferase (CaMKMT-like, At4g35987), which was shown to have a high methylation activity using calmodulin 2 from Arabidopsis as a substrate [56]. The second is an ortholog of prokaryotic PrmA (PrmA-like, At5g53920), the only known Lys-specific protein methyltransferase in bacteria that methylates the ribosomal protein L11 [54].

**Table 1:** Inventory of Arabidopsis PRMT and PKMTs known or predicted to be targeted to chloroplasts.

Gene	Common name	SUBA3		AtSubP	ChloroP 1.1	Experimental localization	References
		consensus	#plastid		(cTP)		
**PRMT**							
AT4G16570	PRMT7	plastid	11	chloroplast	Yes	-	-
**SET-domain PKMTs**							
AT1G14030	LSMT-like	plastid	15	chloroplast	Yes	plastid (MS)	[30], [36], [61]
AT1G24610	PPKMT2	plastid	8	chloroplast	-	-	-
AT3G07670	PPKMT3	plastid	8	chloroplast	Yes	-	-
AT4G20130	PTAC14	plastid	12	chloroplast	Yes	plastid (MS)	[35], [36]
AT5G09790	ATXR5	nucleus	8	nucleus	Yes	nucleus; plastid (GFP)	[64]
AT5G14260	PPKMT1	plastid	9	chloroplast	Yes	plastid (MS)	[36], [61]
**Putative seven-beta-strand PKMTs**							
AT4G35987	CaMKMT-like	plastid	9	chloroplast	Yes	-	-
AT5G53920	PrmA-like	cytosol, mito	9	chloroplast	Yes	-	-

Potential protein Lys/Arg methyltransferases found in the Arabidopsis genome were analyzed for their predicted subcellular localization by using SUBA3, AtSubP, and ChloroP 1.1 [44]–[46]. Proteins with a confident prediction for plastid targeting are shown. Data extracted from the predictors are: for SUBA3, consensus localization and number of predictors (out of 18) indicating a plastid targeting; for AtSubP, prediction using the best hybrid-based classifier (AA+PSSM+N-Center-C+PSI-BLAST); for ChloroP 1.1, presence of a predictable chloroplast transit peptide (cTP). Experimental localizations using GFP-tagging or mass spectrometry (MS) have been reported in the indicated references.

### MS-based identification of Lys- and Arg-methylproteins in Arabidopsis chloroplasts

**Figure 2:**
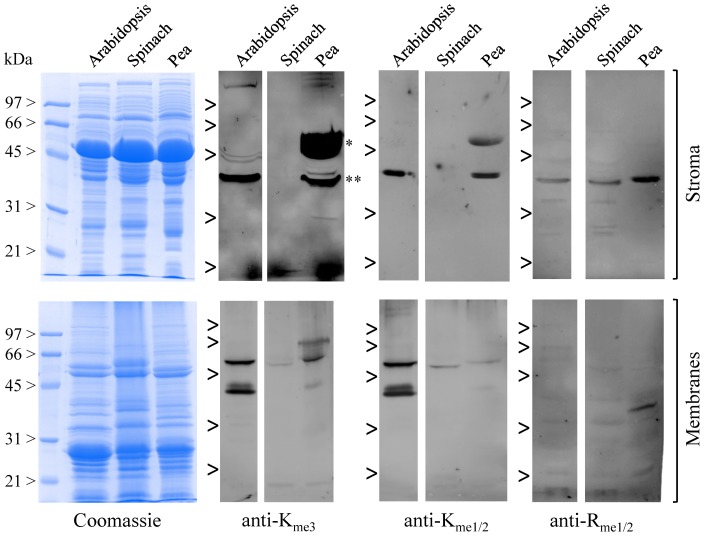
Immunodetection of Lys- and Arg-methylated proteins in chloroplast stroma and membranes subfractions. Chloroplasts from Arabidopsis, spinach and pea leaves were purified using Percoll gradients and fractionated into soluble (stroma) and membrane (thylakoids and envelope) fractions. Fifty µg of proteins were analyzed by SDS-PAGE (Coomassie blue staining) and immunoblotting with antibodies against trimethyl-Lys (anti-K_me3_), mono- and dimethyl-Lys (anti-K_me1/2_), or mono- and dimethyl-Arg (anti-R_me1/2_). The major polypeptides detected by the anti-K_me3_ antibodies are RbcL (*) and fructose bisphosphate aldolases (**) [30].

Although *in vitro* radiolabelling studies using purified organelles were useful to get a first image of the protein-methylating capacity of chloroplasts [25], [26], the approach displayed many drawbacks and did not allow to identify new methylproteins or to assign methylation sites. To address these limitations a common strategy for detection and identification of methylproteins relies on the use of antibodies that are specific to the modified residues and their degree of methylation, with no dependence on surrounding residues [43]. To get an overview of the diversity of Lys- and Arg-methylated proteins in chloroplasts, we performed a set of western blot analyses using soluble (stroma) and membranes (thylakoids plus envelope) subfractions from spinach, pea and Arabidopsis chloroplasts purified on Percoll gradients. Proteins resolved by SDS-PAGE were probed using commercial antibodies specific to mono- and dimethyl-Lys (anti-K_me1/2_), trimethyl-Lys (anti-K_me3_), or mono- and dimethyl-Arg (anti-R_me1/2_). Overall these analyses detected a few Lys- and Arg-methylated proteins split between the soluble and membrane subfractions of chloroplasts (Fig. 2). In stromal fractions, the most immunoreactive bands detected with the anti-K_me3_ antibodies are RbcL (about 53–55 kDa) and aldolases (about 38 kDa) [30]. The limited number of additional candidates could be interpreted as either a low occurrence or high turnover of the modification in chloroplasts and/or a weak performance (sensitivity and specificity) of the antibodies [43].

To overcome the limitation of available antibodies to perform enrichment of methyl-proteins from chloroplast extracts, we developed a MS-based strategy for the identification of authentic Lys- and Arg-methylated proteins on a proteome-wide scale. For this purpose we set up a filtering pipeline for the discovery of Lys- and Arg-methylated peptides in the large pool of spectra collected to edit the Arabidopsis chloroplast proteome (AT_CHLORO database) [36], [37]. AT_CHLORO is a comprehensive database that identifies 1,323 chloroplastic proteins and provides accurate sub-plastidial localization (stroma, envelope, and thylakoids) for 819 proteins. The strategy of reexamination of high-quality MS/MS data using criteria specific to post-translational modifications has been used success-fully to discover methylated proteins in the yeast proteome [11]. The workflow is composed of six steps combining automatic filtering and expert examination of spectra. The procedure is detailed in the Experimental procedure section (Fig. 1). Some of the essential steps to produce high-confidence methylpeptides include the removal of ambiguities due to amino acid substitutions and trimethylation vs. acetylation. These modifications on Lys residues induce very close mass shifts compared to the non-modified peptide (42.04695 and 42.01056 Da, respectively). Instruments with high-mass accuracy, such as the Fourier transform ion cyclotron resonance mass spectrometer used to generate the AT_CHLORO database [36], and identification of neutral loss of trimethylamine (59 Da) that is specific to trimethylation can allow to discriminate these modifications [42], [43]. Nine ambiguous Lys sites were examined and trimethylation was supported for four of them by their accurate mass and the presence of neutral loss at 59 Da (Fig. S1). For one candidate, a glyceraldehyde 3-phosphate dehydrogenase protein (At1g12900), the absence of neutral loss suggested an acetylation, in agreement with a previous proteome survey [21]. This protein was thus eliminated whereas four additional sites belonging to four proteins were retained in the main list since the trimethylation/acetylation ambiguity could not be solved.

The overall filtering pipeline produced a list of 50 different peptide sequences validated from the mass spectrometry aspect and representative of 37 methylated sites (30 Lys and 7 Arg) in 28 non-homologous proteins (Tables 2 and S2). This list is referred to as AT_CHLORO_Me. Among the 37 Lys and Arg methylation sites, four have been previously identified in plant methylproteins, thus validating our approach. Indeed, we identified Lys-methylated peptides originating from aldolase isoforms (At2g21330, At4g38970), as described earlier in Arabidopsis chloroplasts [30]. Also, we identified Arabidopsis PRPL11 (At1g32990) with a trimethylation status at Lys109, a residue that was previously shown to be modified in spinach chloroplasts [31]. Last, our findings confirmed two previously reported methylation sites at Lys44 and Lys187 of the elongation factor 1-alpha (eEF-1A, At1g07920) in Arabidopsis and maize [22], [66].

**Table 2:** Main properties of Lys and Arg methylproteins and methylation sites from the AT_CHLORO_Me inventory.

Protein accession	Symbol	Description	Functional category (MapMan)	Curated location	Methylation sites (status)	3D positioning methylation sites
**Chloroplast-localized proteins**						
AT1G56190.1	PGK2	Phosphoglycerate kinase family protein	Photosynthesis. Calvin cycle	Stroma	K329 (me2)	Surface exposed
AT1G67090.1	RBCS1A	Rubisco small subunit 1A	Photosynthesis. Calvin cycle	Stroma	K66 (me1/2)	Surface exposed
					K140 or K147 (me2)	Surface exposed
					K146 (me1/2)	Surface exposed
AT2G21330.1	FBA1	Fructose-bisphosphate aldolase 1	Photosynthesis. Calvin cycle	Stroma	K395 (me3)c	Protruding
AT2G39730.1	RCA	Rubisco activase	Photosynthesis. Calvin cycle	Stroma	K204 (me1/3)	Surface exposed
AT3G04790.1	PRI	Ribose 5-phosphate isomerase	Photosynthesis. Calvin cycle	Stroma	K200 (me1)	Protruding
AT3G12780.1	PGK1	Phosphoglycerate kinase 1	Photosynthesis. Calvin cycle	Stroma	K424 (me1)	Protruding
ATCG00490.1	RBCL	Rubisco large subunit	Photosynthesis. Calvin cycle	Stroma	K32 (me2/3)a	Protruding
					R79 (me2)	Protruding
					K201 (me1/2/3)	Buried
					K236 (me1/3)a	Buried
					R339 (me1/2)	Surface exposed
					K356 (me2)	Protruding
AT1G32990.1	PRPL11	Plastid ribosomal protein L11	Protein synthesis	Stroma	K109 (me3)c	Protruding
AT1G79530.1	GAPCP1	Glyceraldehyde-3-phosphate dehydrogenase of plastid 1	Glycolysis	Stroma	R407 (me1)	Buried
AT3G11630.1	PrxA	2-Cys peroxiredoxin	Redox	Stroma	K202 (me1/2/3)	Surface exposed
AT5G09650.1	PPA1	Pyrophosphate phospho-hydrolase 1	Nucleotide metabolism	Stroma	K118 (me1)	Buried
AT2G20260.1	PSAE-2	Photosystem I subunit E-2	Photosynthesis. Light reaction	Thylakoid	K145 (me1)	Protruding
AT3G50820.1	PSBO-2	Photosystem II subunit O-2	Photosynthesis. Light reaction	Thylakoid	K292 (me1)	Surface exposed
AT4G17600.1	LIL3∶1	Chlorophyll A-B binding family protein	Photosynthesis. Light reaction	Thylakoid	K101 (me1)	NA
AT4G32260.1	ATPG	ATP synthase beta chain	Photosynthesis. Light reaction	Thylakoid	K129 (me1/3)	NA
ATCG00120.1	ATPA	ATP synthase subunit alpha	Photosynthesis. Light reaction	Thylakoid	R141 (me1)	Surface exposed
ATCG00480.1	ATPB	ATP synthase subunit beta	Photosynthesis. Light reaction	Thylakoid	R52 (me1/2)	Surface exposed
					K447 (me1/2)	Surface exposed
AT1G03630.2	PORC	Protochlorophyllide reductase C	Tetrapyrrole synthesis	Thylakoid	K87 (me2)	Protruding
AT1G06950.1	TIC110	Translocon at the inner envelope membrane of chloroplasts 110	Protein targeting	Envelope	K946 (me2)	NA
AT3G18890.1	TIC62	Translocon at the inner envelope membrane of chloroplasts 62	Protein targeting	Envelope	K79 (me3)a	NA
AT4G13010.1	ceQORH	Quinone-oxidoreductase homolog	Miscellaneous enzyme	Envelope	R119 (me2)	Surface exposed
AT5G42480.1	ARC6	Protein Accumulation and Replication of Chloroplast 6	Cell. Division	Envelope	R516 (me1)	NA
AT5G46110.3	TPT	Triose phosphate/phosphate translocator	Transport	Envelope	K96 (me1)	NA
**Non-chloroplastic proteins**						
AT1G07920.1	eEF-1A	Elongation factor 1-alpha	Protein synthesis	Cytosol	K44 (me1/2)	Protruding
					K187(me3)c	Protruding
AT1G15780.1	MED15A	Mediator of RNA polymerase II transcription subunit 15a	Not assigned	Nucleus	K85 (me1)	NA
AT2G33090.1		Transcription elongation factor (TFIIS) family protein	Not assigned	Nucleus	K79 (me3)c	NA
AT3G48250.1	BIR6	Pentatricopeptide repeat-containing protein	Not assigned	Mitochondrion	K394 (me1)	Surface exposed
AT5G39410.1	SDH	Saccharopine dehydrogenase	Not assigned	Mitochondrion	K6 (me3)a	NA

Protein accession, symbol and description were from TAIR, Uniprot and PPDB databases. Functional annotation was done according to MapMan bins and sub-bins [67]. Subcellular and subplastidial location was curated using data from AT_CHLORO [36], PPDB [62] and dedicated studies. The trimethylated vs. acetylated status of Lys residues was confirmed (c) or remained ambiguous (a). Modeling of 3D-structures was done using the Phyre^2^ server [52]. NA, not available (the protein structure could not be modeled or the model does not cover the methylation site).

### Features and functional analysis of the identified chloroplastic methylproteins

**Figure 3:**
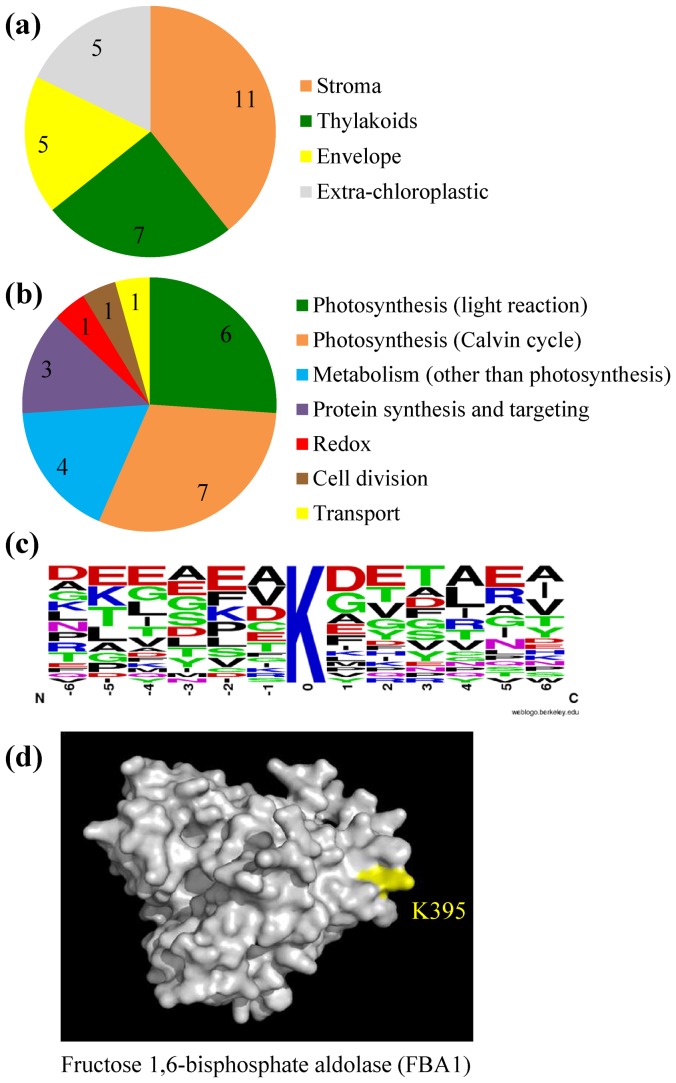
Main features of the identified chloroplastic methylproteins and methylation sites. (a) Curated subcellular/subplastidial location of methylproteins (as in Table 2). (b) Functional categories of chloroplastic methylproteins. Annotated proteins from Table 2 were grouped to create categories ‘metabolism (other than photosynthesis)’ and ‘protein synthesis and targeting’. (c) Amino acid motif surrounding Lys methylation sites was created using WebLogo (http://weblogo.berkeley.edu/). (d) Positioning of the Lys395 methylation site on the 3D-structure model of fructose bisphosphate aldolase (FBA1, At2g21330). The model was generated with the Phyre^2^ server using the 3D structure of aldolase from rabbit muscle (PDB entry 1ZAI) and imaged with the PyMOL software.

Most methylproteins (23 out of 28) from the AT_CHLORO_Me list have been previously identified as authentic chloroplastic proteins in diverse proteomic surveys (Tables 2 and S3). These 23 methylproteins are distributed equally between the stromal soluble phase (11 proteins) and membrane subfractions of chloroplasts (seven in the thylakoids and five in the envelope) (Fig. 3a). The remaining five methylproteins are clearly originating from extra-chloroplastic compartments (cytosol, mitochondrion, and nucleus) and cannot be considered as members of the chloroplastic methylproteome. One example is the Lys-methylated protein eEF-1A that was useful to validate our MS-based approach but is a naturally abundant cytosolic protein often found as a contaminant of organelle proteomes (Table S3).

Methylproteins have been classified according to the MapMan categories to gain insight into their functional implications (Table S3) [67]. Thirteen of the 23 plastidial proteins are involved in photosynthesis (Fig. 3b), with six components of the thylakoid apparatus (e.g. ATP synthase subunits) and seven enzymes of the Calvin cycle (e.g. aldolases). The triose phosphate/phosphate transporter (TPT, At5g46110) could also be connected to this category because it is crucial for the maintenance of high rates of photosynthesis. Other metabolic functions of chloroplasts are also concerned by protein methylation. These include glycolysis (GAPCP1, At1g79530), nucleotide (PPA1, At5g09650) or tetrapyrrole (PORC, At1g03630) synthesis. The remaining five methylproteins have function in the biogenesis and maintenance of chloroplasts (Fig. 3b). They are involved in protein import through the translocon of the inner envelope membrane (TIC62, At3g18890 and TIC110, At1g06950), translation (PRPL11), plastid division (ARC6, At5g42480) or stress signaling (PrxA, At3g11630) (Table 2).

The 23 chloroplastic methylproteins display 24 sites of Lys methylation and 7 sites of Arg methylation. Thus, most of them are modified at a single position, with 16 Lys-methylated and four Arg-methylated proteins (Table 2). The observed Lys- to Arg-methylsites ratio of about 4 is markedly different from those previously detected in cellular methylproteomes from yeast (about 1 to 1 ratio, but K_me3_ were not considered) [11] or human cells (18% Lys-methylsites) [13]. The prevalence of methyl-Lys in chloroplasts is in accordance with the relative number of chloroplastic PKMTs (8 proteins) and PRMT (1 protein) identified in these organelles (Table 1) and is likely physiologically relevant.

It is well established that the sub-stoichiometric occupancy and the possible reversible nature of some methylation sites add substantially to the complexity of identifying methylproteins [42], [43]. To have an estimate of the overall methylation status of the identified proteins we used spectral counts (SC) as rough indicators of peptides abundance and compared values for methylated peptide sequences with total SC values covering the sites, i.e. methylated and unmodified peptides (Table S2). Ten methylation sites were estimated to be fully or almost fully modified; they belong to 10 different proteins including aldolases or PRPL11. Methylation at the other sites was incomplete, with SC ratio in favor of unmodified sites (e.g. Rubisco activase and some RbcL methylation sites). Thus, our approach was successful for the identification of methylproteins with very distinct levels of methylation *in vivo*.

We analyzed the list of Lys methylation sites for sequence composition proximal (±6 residues) from the modification site. This analysis did not reveal any significant consensus motif around methyl-Lys (Fig. 3c), a feature that was already noticed in the human cellular methylproteome [13] and in bioinformatic analyses gathering a large number of sites [49], [50]. The absence of motif suggested that chloroplastic PKMTs (Table 1) recognize distinct peptide sequences and display different substrate specificities. The very limited number (seven) of identified methyl-Arg sites precluded any interpretation about the specificity of protein Arg methylation in chloroplasts. To assess whether the identified methylation sites in chloroplastic proteins shared features other than simple sequence motifs with previously characterized methylproteins, we submitted each protein sequence to a range of publicly available tools. These are designed to predict methylation sites using several features including secondary structure or physicochemical properties of residues [49], [50]. This analysis gave only few matches between experimental and predictive sites (Table S3), pointing out the limitation of currently available algorithms to identify Lys/Arg methylproteins [68], at least in chloroplasts.

To get insight into the functional significance of methylation we investigated the position of the identified Lys/Arg residues within the three-dimensional structures of methylproteins. Thus, we generated 3D models using structural data of homologous proteins that share a high sequence similarity with the respective Arabidopsis proteins. Eighteen out of 23 proteins could be satisfactorily modeled using the Phyre^2^ webserver [52], giving position information for 25 methylation sites. In most cases, modified residues were predicted to be surface-exposed or protruding the surface (Fig. 3d) and very few (4 sites) were buried inside the protein cores (Table 2, Fig. S2). Therefore, the large majority of the identified methylation sites is expected to be freely accessible to protein Lys/Arg methyltransferases. Modification of hidden sites may possibly occur before final protein folding, e.g. following the import step through the chloroplast envelope for proteins encoded by the nuclear genome, or may result from a dynamic flexibility of the protein enabling the residue to flip out of the structure for the methylation reaction [1]. Also, the location of methylated sites to surface-exposed regions of targets suggests possible roles in protein-protein interactions, Lys/Arg methylation being recognized as a key regulatory element in the modulation of macromolecular interactions [3], [6]. To support this assumption a review of the Arabidopsis interactome map [69] identified 12 chloroplastic proteins of AT_CHLORO_Me involved in binary protein-protein interactions. For example, the yeast two-hybrid system and literature-curated binary interactions identified the chlorophyll-synthesizing enzyme PORC (At1g03630), the TIC110 component of the translocon at the chloroplast inner envelope (At1g06950), and the ARC6 protein (At5g42480) that is involved in the assembly of the plastid-dividing FtsZ ring. Other studies have previously shown that chloroplastic methylproteins are components of transient or stable complexes, e.g. fructose bisphosphate aldolase [70] or PRPL11 [31]. Together, these data suggest that Lys/Arg methylation may modulate protein-protein interactions involved in chloroplast biogenesis (protein import, plastid division, translation) and metabolism (photosynthesis, chlorophyll synthesis).

### Biochemical analysis of some methylproteins

**Figure 4:**
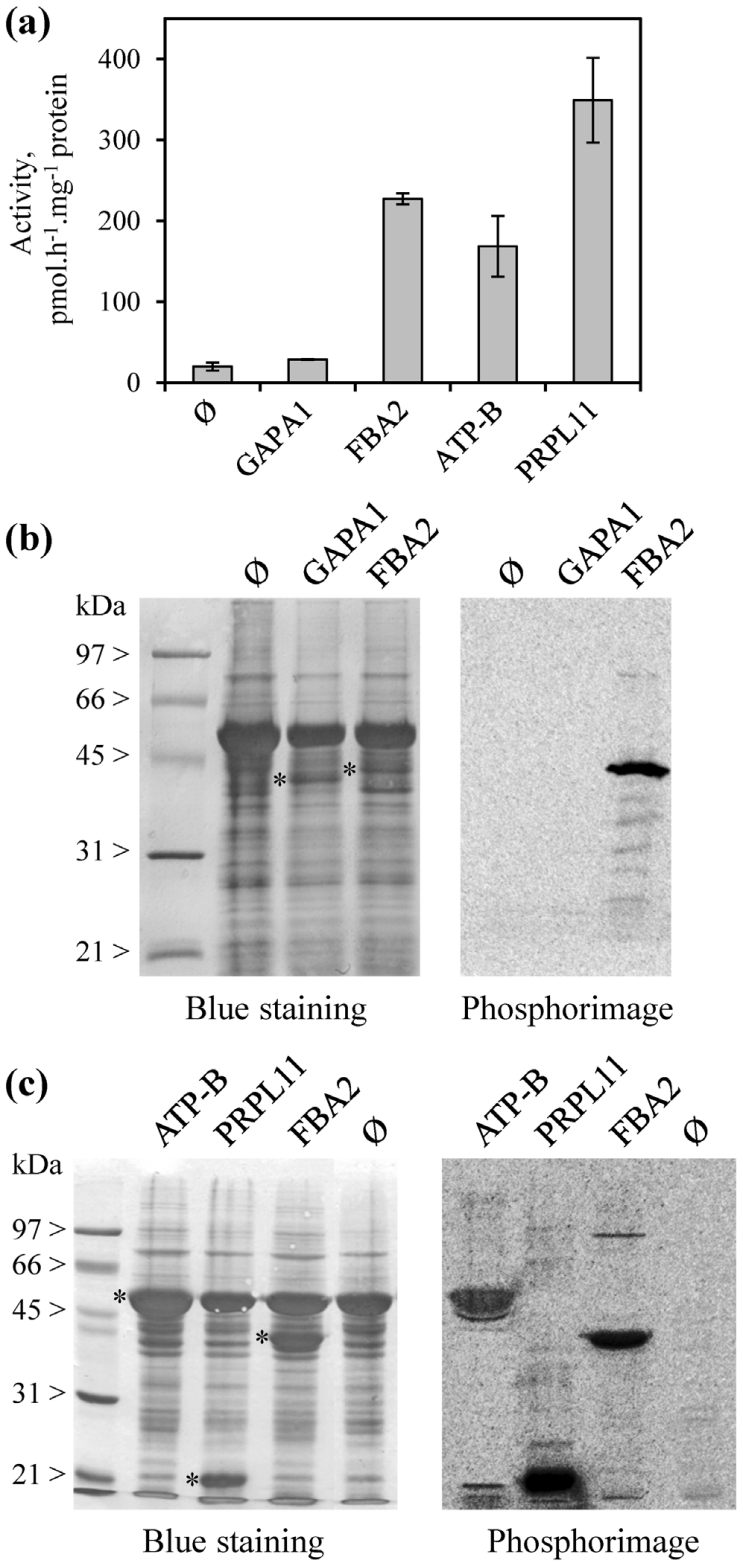
Biochemical validation of methylproteins through *in vitro* methylation assays using stroma from Arabidopsis chloroplasts. Methylation assays were done in the presence of 20 µg purified recombinant targets (FBA2, GAPA1, PRPL11, or ATP-B), 80 µg stroma from Arabidopsis Col-0 chloroplasts, 20 µM [methyl-^3^H]-AdoMet and 100 nM *S*-adenosylhomocysteine hydrolase. After incubation at 30°C for 1 to 2 hours, assays were split into two equals parts and radioactivity incorporated into proteins was counted by liquid scintillation (panel a) and analyzed by phosphorimaging (panels b–c). In panels (a–c), the symbol Ø means that no recombinant protein was added to the stromal extract. Purified recombinant substrates are indicated by asterisks: ATP-B, 54 kDa; FBA2, 40 kDa; GAPA1, 38 kDa; PRPL11, 18 kDa (Fig. S3). Activities with the FBA2, PRPL11 and ATP-B substrates were strictly dependent on the addition of stroma. Values are mean ± SD of two to six independent determinations.

Reconstitution of the methyltransferases/methylproteins relationships using the established lists of potential enzymes and substrates (Tables 1 and 2) is necessary to gain insight into the role of protein methylation in chloroplasts. As a first step towards building this network, we produced several identified targets as recombinant proteins in *Escherichia coli* for *in vitro* methylation experiments with chloroplast stroma. Expressed proteins were fructose bisphosphate aldolase (FBA2, At4g38970), a previously validated methylprotein [30], the β-subunit of ATP synthase (ATP-B; AtCg00480), PRPL11 (At1g32990), and glyceraldehyde 3-phosphate dehydrogenase (GAPA1, At3g26650), an ambiguous candidate for which modification at Lys311 was attributed to acetylation rather than trimethylation (Fig. S1). Recombinant proteins were purified (Fig. S3) and further used for *in vitro* methylation assays using [methyl-^3^H]-AdoMet and chloroplast stroma from wild-type Arabidopsis as a source of methyltransferases (chloroplastic PKMTs and PRMT7 are stromal proteins or predicted to be soluble). As shown in Fig. 4a,b, significant stromal-dependent methyl-group incorporation was observed for the positive control FBA2 whereas GAPA1 was not methylated, meaning that the assay was suitable to follow methylation of true substrates. The assay also validated the removal of GAPA1 from the set of chloroplastic methyl-proteins. The other two proteins PRPL11 and ATP-B were significantly methylated (Fig. 4a,c), suggesting strongly that they are authentic methylproteins modified by stromal methyl-transferases.

To confirm the methylated sites on recombinant substrates, *in vitro* methylation reactions were repeated with unlabeled AdoMet. Target proteins were submitted to LC-MS/MS after tryptic cleavage and spectra were analyzed using the methyl-search parameters. For PRPL11, Lys109 was found unmodified in the control experiment without AdoMet and peptides bearing a K_me3_ at position 109 were identified in the complete assay mixture (Fig. S4). These results indicated that a chloroplastic PKMT was efficient to modify this site *in vitro* in our assay conditions.

Methylation assays also enabled us to show that Lys447 of recombinant ATP-B is methylatable by a chloroplastic enzyme. Indeed, the complete assay resulted in dimethylation at Lys447 whereas this position was not modified in the untreated recombinant protein (Fig. S4). Arg52 was identified as a second methylation site of ATP-B but we could not methylate this site by the *in vitro* approach. It should be reminded that the methylation ratio observed at this site *in vivo* was largely in favor of the unmodified residue (2 methylated peptides over 45 covering the site; Table S2). Thus, it is likely that the enzymatic assay was unsuitable to reproduce this situation, possibly because of a low activity and/or stability of the associated PRMT in chloroplast stroma (ATP-B cannot be methylated outside from chloroplasts because it is encoded by the plastid genome).

### Identification of chloroplastic PKMTs involved in the methylation of PRPL11 and ATP-B

**Figure 5:**
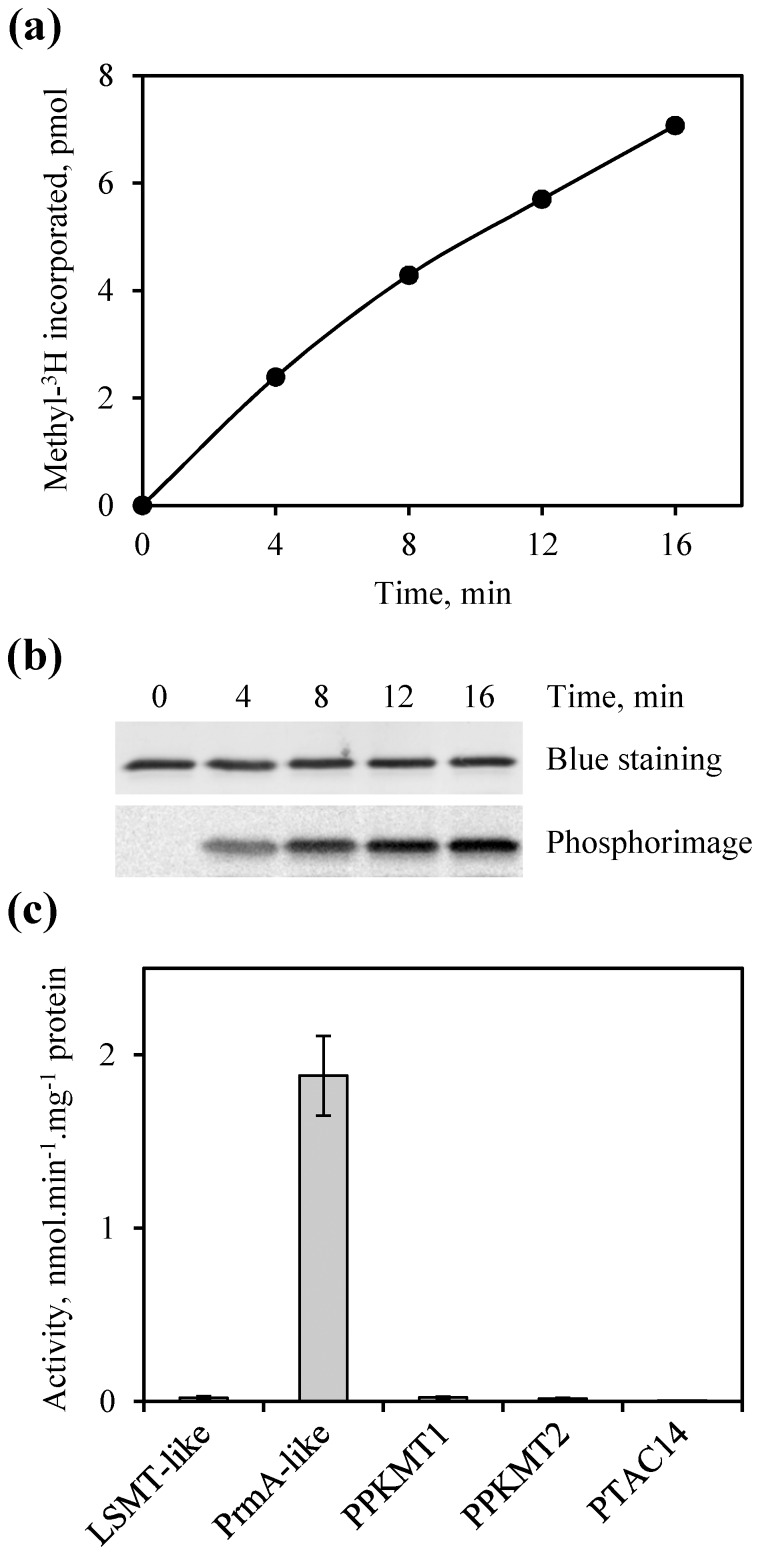
*In vitro* methylation of PRPL11 by PrmA-like. (a,b) Kinetic analysis of PRPL11 methylation by PrmA-like. Purified recombinant PRPL11 (2.5 µg) and PrmA-like (0.25 µg) were incubated at 30°C with 20 µM [methyl-^3^H]-AdoMet. Radioactivity incorporated into proteins was counted by liquid scintillation (a) or analyzed by phosphorimaging (b). A time-course analysis representative of three repeats is shown. The activity (2.0±0.2 nmol methyl incorporated. min^−1^.mg^−1^ protein) was strictly dependent on the presence of PrmA-like. (c) Screening of five chloroplastic PKMTs for their ability to methylate PRPL11. Purified recombinant PRPL11 (10 µg) and PKMTs (1 µg) were incubated at 30°C for 15 min with 20 µM [methyl-^3^H]-AdoMet and radioactivity incorporated into proteins was counted by liquid scintillation. Enzyme nomenclature is as in Table 1. Values are mean ± SD of three determinations.

We further tested the usefulness and robustness of our inventory of PKMTs (Table 1) to reconstitute enzyme/substrate relationships. A confrontation of the set of chloroplastic methyltransferases and methylproteins with the literature suggested strongly that PRPL11 could be the substrate of PrmA-like, PrmA being responsible for RPL11 methylation in bacteria [54]. To test this hypothesis, we purified a recombinant Arabidopsis PrmA-like protein produced in *E. coli* (Fig. S3) and performed methylation assays in the presence of recombinant PRPL11 and [methyl-^3^H]-AdoMet. As shown in Fig. 5a,b, kinetic analysis resulted in linear incorporation of methyl-groups into PRPL11. Also, sequencing of the methylated product by MS/MS indicated that trimethylation occurred at Lys109, as for the assays conducted with stroma, validating PRPL11 as a substrate of PrmA-like. To analyze whether functional redundancy can exist among protein methyltransferases, we tested the ability of four alternative chloroplastic PKMTs to modify PRPL11 *in vitro*. LSMT-like (At1g14030) and PPKMT1 (At5g14260) had been purified previously from bacterial overproducing strains [30]. LSMT-like was shown to methylate aldolases whereas PPKMT1 had no activity with these substrates. The PTAC14 (At4g20130) and PPKMT2 (At1g24610) proteins were also produced in *E. coli* and purified as recombinant proteins (Fig. S3). Among the five PKMTs tested, PrmA-like was the only capable to methylate PRPL11 (Fig. 5c). These data suggested that methylation of the protein L11 component of ribosomal 50S subunit by the seven-beta-strand enzyme PrmA is an evolutionary conserved process from bacteria [54] to higher plants.

**Figure 6:**
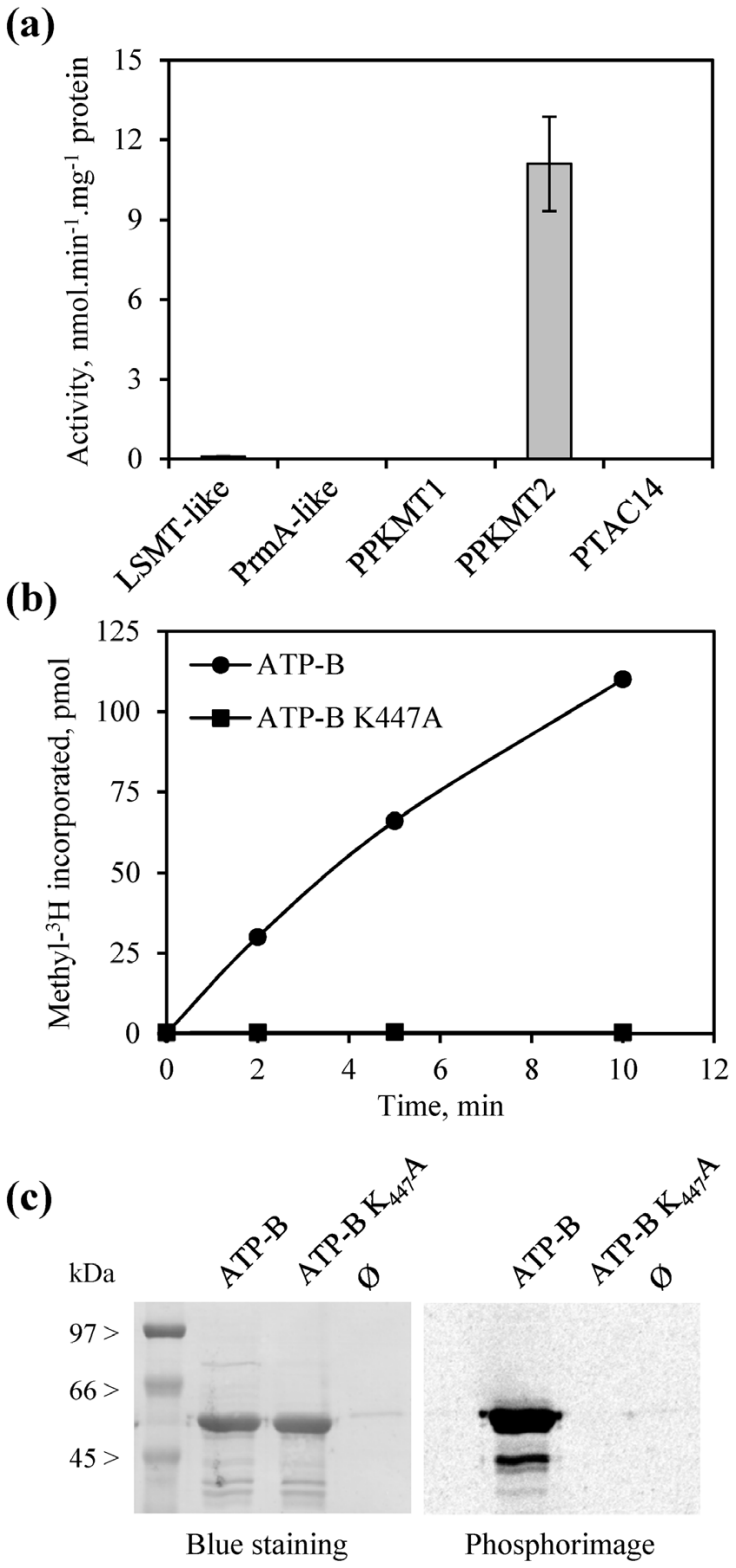
*In vitro* methylation of the ATP synthase β-subunit by PPKMT2. (a) Screening of five chloroplastic PKMTs for their capacity to methylate ATP-B. Purified recombinant ATP-B (24 µg) and PKMTs (1 µg) were incubated at 30°C for 10 min with 20 µM [methyl-^3^H]-AdoMet and radioactivity incorporated into proteins was counted by liquid scintillation. Enzyme nomenclature is as in Table 1. Values are mean ± SD of two to four independent determinations. (b) Time-course analysis of ATP-B methylation by PPKMT2. Assays were as in (a) and included the wild-type version of ATP-B or the K_447_A mutant. Kinetic analyses representative of three repeats are shown (11.1±1.7 nmol methyl incorporated.min^−1^.mg^−1^ protein with the ATP-B substrate). (c) Phosphorimage analysis of ATP-B methylation by PPKMT2. ATP-B (10 µg) and PPKMT2 (0.3 µg) were incubated at 30°C for 10 min with 20 µM [methyl-^3^H]-AdoMet. Symbol Ø means that no protein substrate was included in the assay.

The present work is the first report that describes ATP-B from thylakoids as a Lys-methylated protein, thus precluding identification of the associated methyltransferase by homology. To identify this enzyme we screened purified chloroplastic PKMTs for their ability to methylate recombinant ATP-B *in vitro*. As shown in Fig. 6a, PPKMT2 was the only enzyme capable of transferring methyl-groups on the ATP-B substrate. To ascertain that modification of ATP-B by PPKMT2 occurred at the expected lysyl residue we produced and purified an ATP-B mutant bearing a substitution of Lys447 by a non-methylatable alanine (ATP-B K_447_A) and used this substrate for *in vitro* methylation assays. As shown in Fig. 6b,c, methylation of ATP-B by PPKMT2 was fully abolished with the ATP-B K_447_A mutant, demonstrating that the modification is specific for Lys447. Together, these data demonstrated that the thylakoid-associated protein ATP-B can be methylated by the soluble enzyme PPKMT2.

## Conclusion

This study describes the first proteome-wide identification of non-histone Lys and Arg methylated proteins in a photosynthetic organism together with the inventory of the methyl-transferases potentially involved in their modification. The present reference map of methylproteins suggests that methylation may have a significant role in chloroplast biology. Indeed, the identified methylproteins are involved in a variety of metabolic pathways and processes distributed over the three main chloroplast sub-compartments. Through the identification of a set of chloroplastic protein methyltransferases, our data also provide a foundation to build the enzymes/substrates relationships that govern methylation in the chloroplast. Unraveling this network is a crucial step toward understanding the role of protein methylation in chloroplast biology, which is currently fully unknown. Functional characterization of Arabidopsis lines affected in the expression of chloroplastic methyltransferases will be decisive to achieve this goal. Also, knowing the position and methylation status of Lys/Arg sites on methylproteins will provide the opportunity to analyze methylproteome dynamics in response to diverse developmental and environmental signals, giving insights into the regulatory (dynamic) or structural (static) role of the modification.

## Supporting Information

Figure S1 Discrimination between Lys trimethylation and acetylation in ambiguous candidates.
(PDF)

Figure S2 Positioning of methylation sites on the 3D structure models of some identified methylproteins.
(PDF)

Figure S3 Documentation on recombinant protein substrates and methyltransferases used for *in vitro* methylation assays.
(PDF)

Figure S4 LC-MS/MS fragmentation spectra of recombinant PRPL11 and ATP-B methylated *in vitro* by chloroplast stroma.
(PDF)

Table S1 Oligonucleotides used in this study.
(PDF)

Table S2 MS/MS identification of candidate methylproteins.
(XLS)

Table S3 Features of methylproteins from the AT_CHLORO_Me list.
(XLS)
