# Supplementary material for: Uncovering the Protein Lysine and Arginine Methylation Network in Arabidopsis Chloroplasts
Source: PLoS One. 2014 Apr 18;9(4):e95512. doi: 10.1371/journal.pone.0095512 (PMC3991674; doi:10.1371/journal.pone.0095512)
Supplement: Figure S1 — Discrimination between Lys trimethylation and acetylation in ambiguous candidates. (PDF) [file pone.0095512.s001.pdf]

Supplemental Figure S1: Discrimination between Lys trimethylation and acetylation in ambiguous candidates.

For each site with a trimethyl-Lys to be examined, we considered the PSM of highest score and the corresponding acquisition run.

Summary of the examined ambiguities:

Protein name	TAIR accession	Site	Precursor Error (ppm)	Neutral loss (-59)	Decision
FBA1	AT2G21330.1	K395	1.385	yes	accept Me3
eEF-1A	AT1G07920.1	K187	5.121	yes	accept Me3
GAPA2	AT1G12900.1	K314	-7.962	no	reject Me3
RBCL	AtCg00490	K32	-6.131	yes	ambiguous
RBCL	AtCg00490	K236	-5.208	yes	ambiguous
Tic62	AT3G18890.1	K79	-7.947	yes	ambiguous
SDH	AT5G39410.1	K6	-1.729	yes	ambiguous
PRPL11	AT1G32990.1	K109	7.941	yes	accept Me3
-	AT2G33090.1	K79	5.803	yes	accept Me3

Error distribution graphs: distribution of the mass measurement errors within the acquisition run for all PSMs having a score > 40. Abscissa: error values in ppm; coordinates: number of PSMs.
Blue line: true error for the candidate trimethyl-PSM to be examined.
Red line: artificial error for the same PSM if one substitutes trimethylation for acetylation.

MS/MS spectra: fragmentation spectra and ions tables of trimethyl-PSM candidates extracted from Mascot interpretation.

FBA1, AT2G21330.1

YTGEGESEEAKEGMFV**K**$_{395}$GYTY, expmz 2448.071248

Interpreted with: M14, Dioxidation (M); K17, Trimethyl (K)

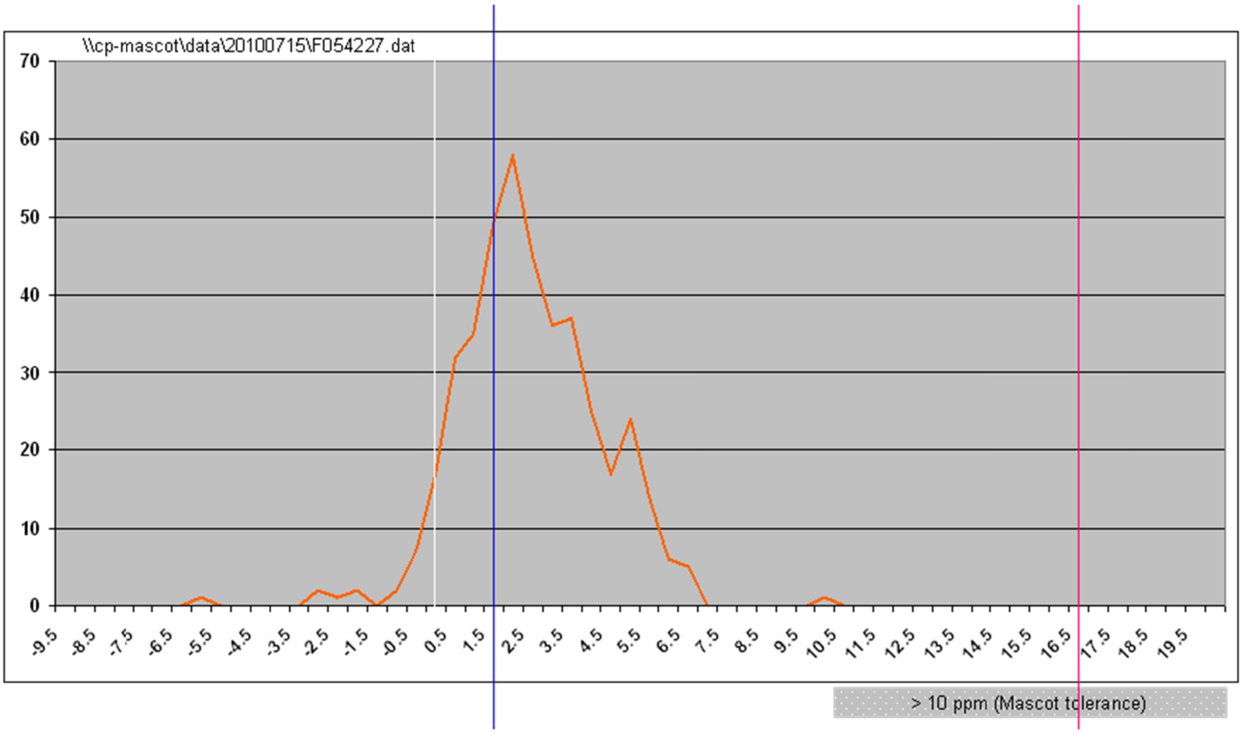

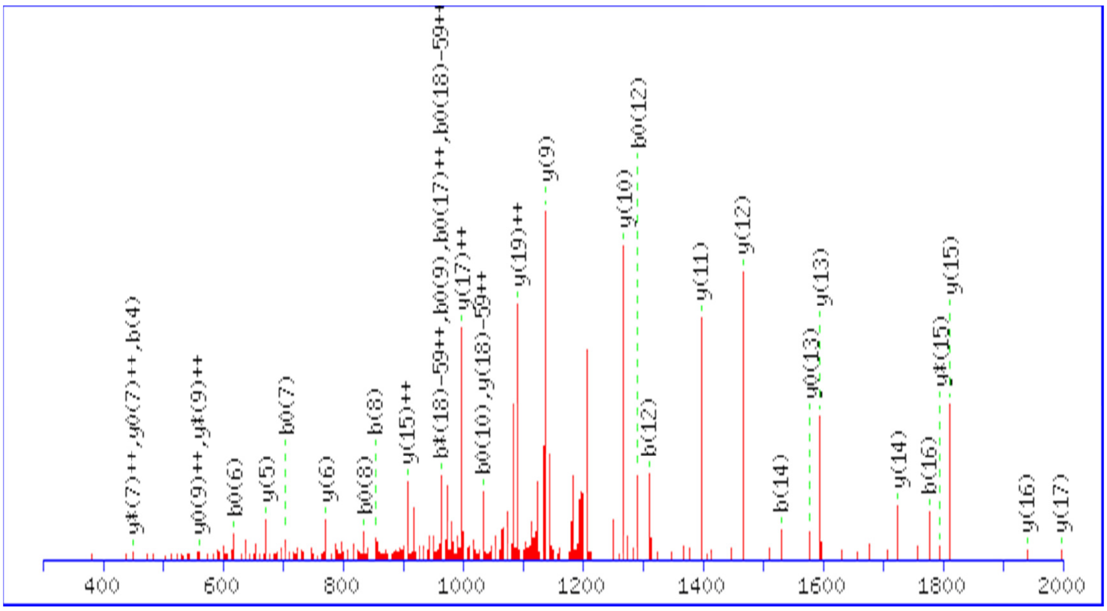

#	b	b^{++}	b*	b^{*++}	b^{0}	b^{0++}	Seq.	y	y^{++}	y*	y^{*++}	y^{0}	y^{0++}	#
1	164.0706	82.5389					Y							21
2	265.1183	133.0628			247.1077	124.0575	T	2286.0118	1143.5096	2268.9853	1134.9963	2268.0013	1134.5043	20
3	322.1397	161.5735			304.1292	152.5682	G	2184.9642	1092.9857	2167.9376	1084.4724	2166.9536	1083.9804	19
4	**451.1823**	226.0948			433.1718	217.0895	E	2127.9427	1064.4750	2110.9161	1055.9617	2109.9321	1055.4697	18
5	508.2038	254.6055			490.1932	245.6003	G	***1998.9001***	999.9537	1981.8736	991.4404	1980.8895	990.9484	17
6	637.2464	319.1268			619.2358	310.1216	E	***1941.8786***	971.4430	1924.8521	962.9297	1923.8681	962.4377	16
7	724.2784	362.6429			706.2679	353.6376	S	***1812.8360***	906.9217	1795.8095	898.4084	1794.8255	897.9164	15
8	**853.3210**	427.1641			835.3105	418.1589	E	***1725.8040***	863.4056	1708.7775	854.8924	1707.7935	854.4004	14
9	982.3636	491.6854			964.3530	482.6802	E	***1596.7614***	798.8844	1579.7349	790.3711	1578.7509	789.8791	13
10	1053.4007	527.2040			1035.3902	518.1987	A	***1467.7188***	734.3631	1450.6923	725.8498	1449.7083	725.3578	12
11	1181.4957	591.2515	1164.4691	582.7382	1163.4851	582.2462	K	***1396.6817***	698.8445	1379.6552	690.3312	1378.6712	689.8392	11
12	**1310.5383**	655.7728	1293.5117	647.2595	1292.5277	646.7675	E	***1268.5868***	634.7970	1251.5602	626.2837	1250.5762	625.7917	10
13	1367.5597	684.2835	1350.5332	675.7702	1349.5492	675.2782	G	***1139.5442***	570.2757	1122.5176	561.7624	1121.5336	561.2704	9
14	**1530.5901**	765.7987	1513.5635	757.2854	1512.5795	756.7934	M	1082.5227	541.7650	1065.4961	533.2517	1064.5121	532.7597	8
15	1677.6585	839.3329	1660.6319	830.8196	1659.6479	830.3276	F	919.4924	460.2498	902.4658	451.7366	901.4818	451.2445	7
16	**1776.7269**	888.8671	1759.7003	880.3538	1758.7163	879.8618	V	***772.4240***	386.7156	755.3974	378.2023	754.4134	377.7103	6
17	1946.8688	973.9380	1929.8423	965.4248	1928.8582	964.9328	K	***673.3556***	337.1814	656.3290	328.6681	655.3450	328.1761	5
18	2003.8903	1002.4488	1986.8637	993.9355	1985.8797	993.4435	G	503.2136	252.1105			485.2031	243.1052	4
19	2166.9536	1083.9804	2149.9270	1075.4672	2148.9430	1074.9752	Y	446.1922	223.5997			428.1816	214.5944	3
20	2268.0013	1134.5043	2250.9747	1125.9910	2249.9907	1125.4990	T	283.1288	142.0681			265.1183	133.0628	2
21							Y	182.0812	91.5442					1

eEF-1A, AT1G07920.1

VGYNPD**K**$_{187}$IPFVPISGFEGDNMIER, expmz 2751.371892

Interpreted with: K7, Trimethyl (K); M21, Oxidation (M)

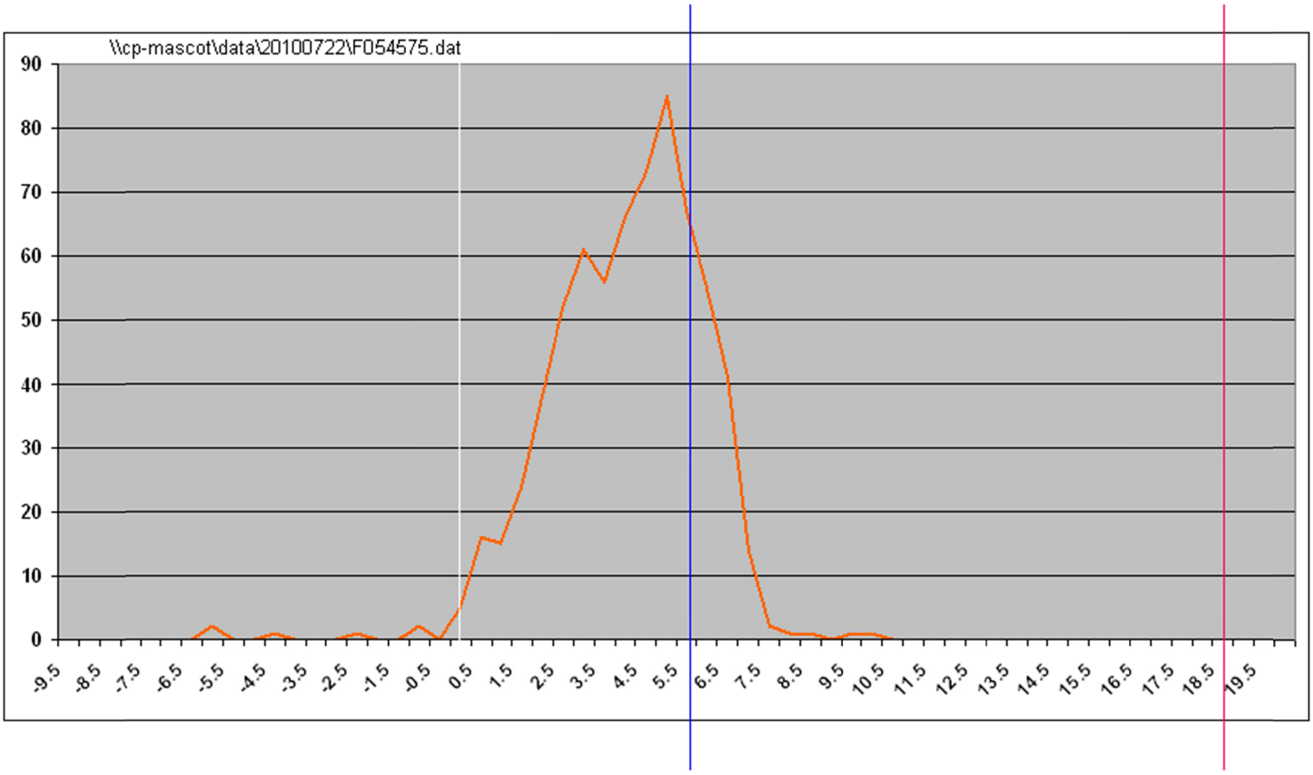

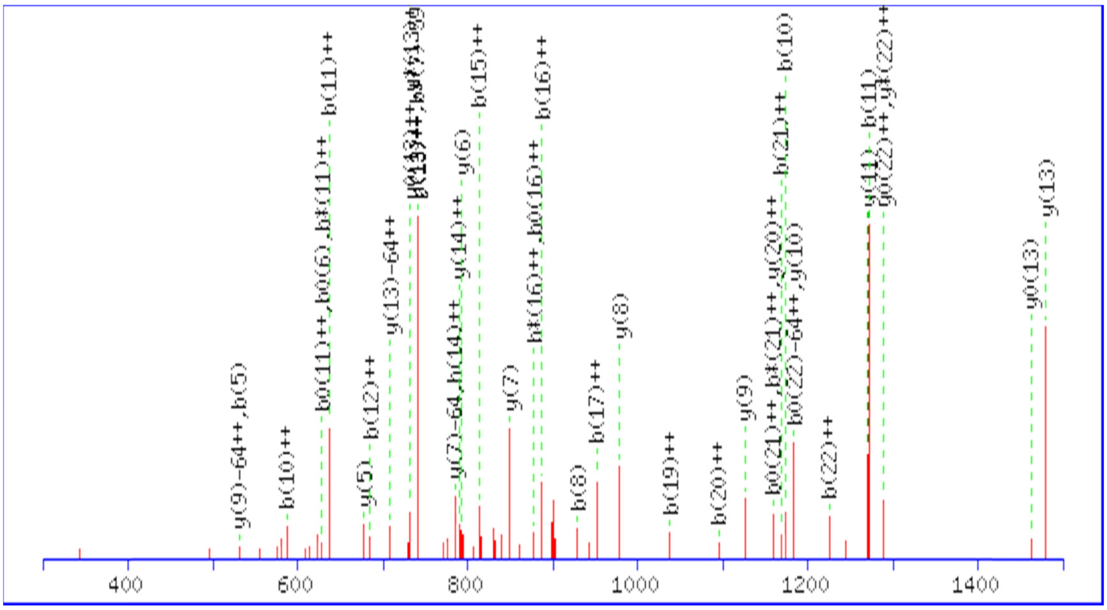

Zoom in the 720-760 ppm range:

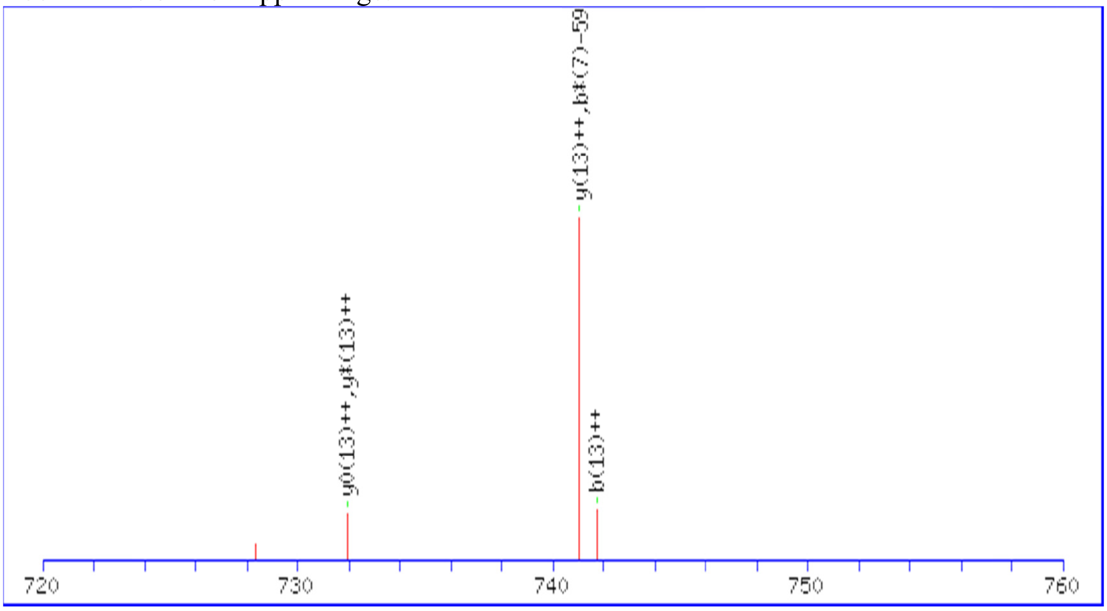

#	b	b^{++}	b*	b^{*++}	b^{0}	b^{0++}	Seq.	y	y^{++}	y*	y^{*++}	y^{0}	y^{0++}	#
1	100.0757	50.5415					V							24
2	157.0972	79.0522					G	2653.2967	1327.1520	2636.2701	1318.6387	2635.2861	1318.1467	23
3	320.1605	160.5839					Y	2596.2752	1298.6412	2579.2487	1290.1280	2578.2646	1289.6360	22
4	434.2034	217.6053	417.1769	209.0921			N	2433.2119	1217.1096	2416.1853	1208.5963	2415.2013	1208.1043	21
5	**531.2562**	266.1317	514.2296	257.6185			P	2319.1689	1160.0881	2302.1424	1151.5748	2301.1584	1151.0828	20
6	646.2831	323.6452	629.2566	315.1319	628.2726	314.6399	D	2222.1162	1111.5617	2205.0896	1103.0485	2204.1056	1102.5564	19
7	816.4250	408.7162	799.3985	400.2029	798.4145	399.7109	K	2107.0892	1054.0483	2090.0627	1045.5350	2089.0787	1045.0430	18
8	**929.5091**	465.2582	912.4825	456.7449	911.4985	456.2529	I	1936.9473	968.9773	1919.9208	960.4640	1918.9368	959.9720	17
9	1026.5619	513.7846	1009.5353	505.2713	1008.5513	504.7793	P	1823.8633	912.4353	1806.8367	903.9220	1805.8527	903.4300	16
10	**1173.6303**	***587.3188***	1156.6037	578.8055	1155.6197	578.3135	F	1726.8105	863.9089	1709.7839	855.3956	1708.7999	854.9036	15
11	**1272.6987**	***636.8530***	1255.6721	628.3397	1254.6881	627.8477	V	1579.7421	790.3747	1562.7155	781.8614	1561.7315	781.3694	14
12	1369.7515	***685.3794***	1352.7249	676.8661	1351.7409	676.3741	P	**1480.6737**	740.8405	1463.6471	732.3272	1462.6631	731.8352	13
13	1482.8355	***741.9214***	1465.8090	733.4081	1464.8249	732.9161	I	1383.6209	692.3141	1366.5944	683.8008	1365.6103	683.3088	12
14	1569.8675	***785.4374***	1552.8410	776.9241	1551.8570	776.4321	S	**1270.5368**	635.7721	1253.5103	627.2588	1252.5263	626.7668	11
15	1626.8890	***813.9481***	1609.8625	805.4349	1608.8784	804.9429	G	**1183.5048**	592.2560	1166.4783	583.7428	1165.4942	583.2508	10
16	1773.9574	***887.4823***	1756.9309	878.9691	1755.9469	878.4771	F	**1126.4834**	563.7453	1109.4568	555.2320	1108.4728	554.7400	9
17	1903.0000	***952.0036***	1885.9735	943.4904	1884.9894	942.9984	E	**979.4149**	490.2111	962.3884	481.6978	961.4044	481.2058	8
18	1960.0215	980.5144	1942.9949	972.0011	1942.0109	971.5091	G	**850.3723**	425.6898	833.3458	417.1765	832.3618	416.6845	7
19	2075.0484	***1038.0278***	2058.0219	1029.5146	2057.0379	1029.0226	D	**793.3509**	397.1791	776.3243	388.6658	775.3403	388.1738	6
20	2189.0913	***1095.0493***	2172.0648	1086.5360	2171.0808	1086.0440	N	**678.3239**	339.6656	661.2974	331.1523	660.3134	330.6603	5
21	2336.1267	***1168.5670***	2319.1002	1160.0537	2318.1162	1159.5617	M	564.2810	282.6441	547.2545	274.1309	546.2704	273.6389	4
22	2449.2108	***1225.1090***	2432.1843	1216.5958	2431.2002	1216.1038	I	417.2456	209.1264	400.2191	200.6132	399.2350	200.1212	3
23	2578.2534	***1289.6303***	2561.2269	1281.1171	2560.2428	1280.6251	E	304.1615	152.5844	287.1350	144.0711	286.1510	143.5791	2
24							R	175.1190	88.0631	158.0924	79.5498			1

GAPA2, AT1G12900.1

VPTPNVSVVDLVVQVS**K**$_{314}$, expmz 1821.046688

Interpreted with: K17, Trimethyl (K)

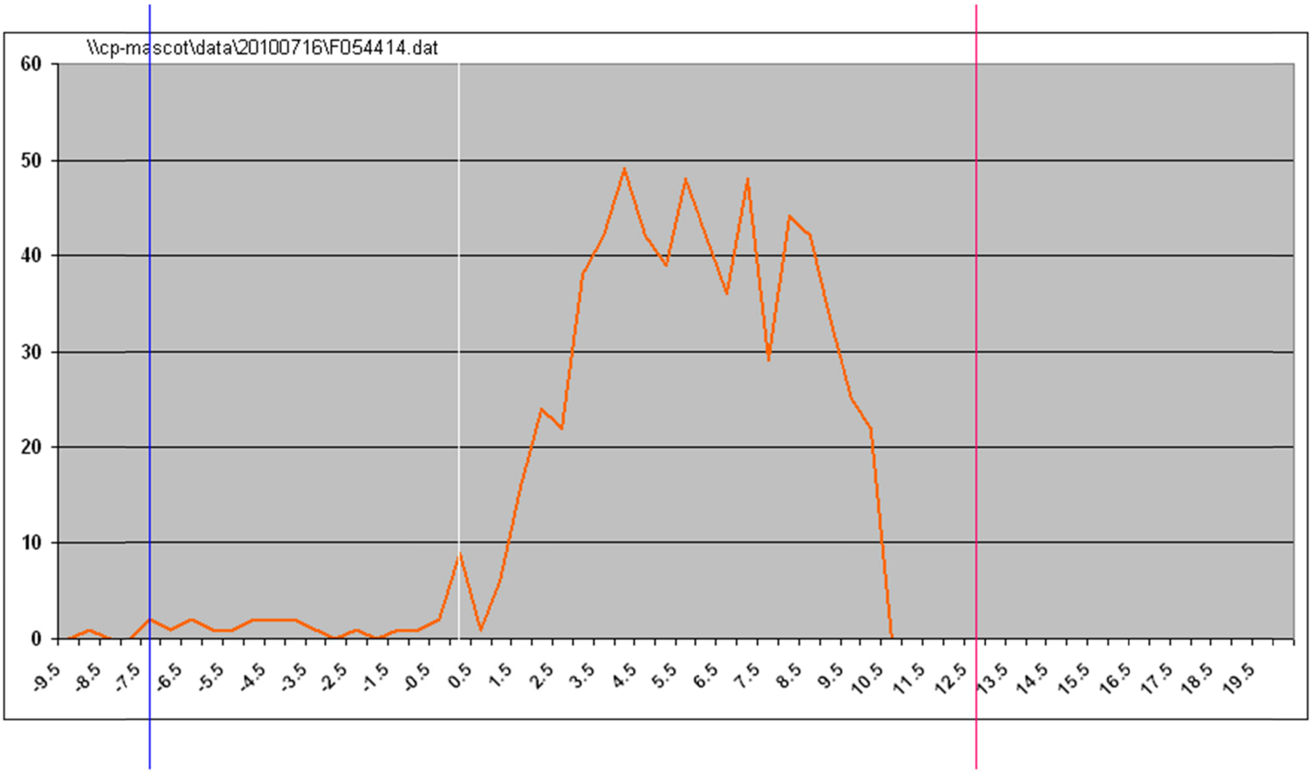

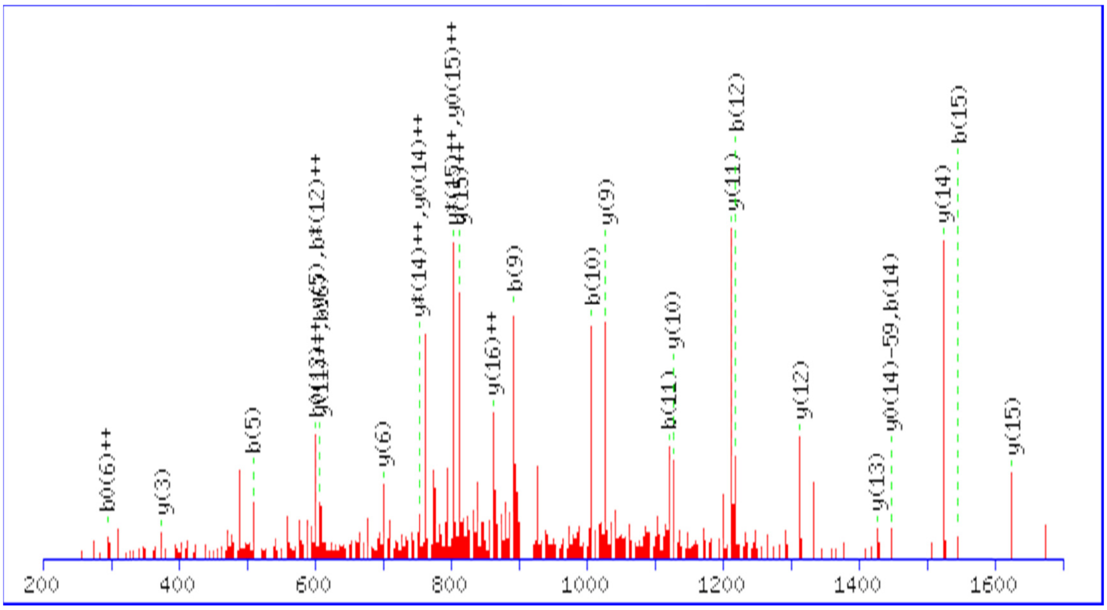

#	b	b^{++}	b*	b^{*++}	b^{0}	b^{0++}	Seq.	y	y^{++}	y*	y^{*++}	y^{0}	y^{0++}	#
1	100.0757	50.5415					V							17
2	197.1285	99.0679					P	1723.0000	862.0036	1705.9735	853.4904	1704.9894	852.9984	16
3	298.1761	149.5917			280.1656	140.5864	T	***1625.9472***	813.4773	1608.9207	804.9640	1607.9367	804.4720	15
4	395.2289	198.1181			377.2183	189.1128	P	***1524.8996***	762.9534	1507.8730	754.4401	1506.8890	753.9481	14
5	***509.2718***	255.1396	492.2453	246.6263	491.2613	246.1343	N	***1427.8468***	714.4270	1410.8203	705.9138	1409.8362	705.4218	13
6	***608.3402***	304.6738	591.3137	296.1605	590.3297	295.6685	V	***1313.8039***	657.4056	1296.7773	648.8923	1295.7933	648.4003	12
7	695.3723	348.1898	678.3457	339.6765	677.3617	339.1845	S	***1214.7355***	607.8714	1197.7089	599.3581	1196.7249	598.8661	11
8	794.4407	397.7240	777.4141	389.2107	776.4301	388.7187	V	***1127.7034***	564.3554	1110.6769	555.8421	1109.6929	555.3501	10
9	***893.5091***	447.2582	876.4825	438.7449	875.4985	438.2529	V	***1028.6350***	514.8211	1011.6085	506.3079	1010.6245	505.8159	9
10	***1008.5360***	504.7717	991.5095	496.2584	990.5255	495.7664	D	929.5666	465.2869	912.5401	456.7737	911.5560	456.2817	8
11	***1121.6201***	561.3137	1104.5936	552.8004	1103.6095	552.3084	L	814.5397	407.7735	797.5131	399.2602	796.5291	398.7682	7
12	***1220.6885***	610.8479	1203.6620	602.3346	1202.6780	601.8426	V	***701.4556***	351.2314	684.4291	342.7182	683.4450	342.2262	6
13	1319.7569	660.3821	1302.7304	651.8688	1301.7464	651.3768	V	***602.3872***	301.6972	585.3606	293.1840	584.3766	292.6919	5
14	***1447.8155***	724.4114	1430.7890	715.8981	1429.8049	715.4061	Q	503.3188	252.1630	486.2922	243.6498	485.3082	243.1577	4
15	***1546.8839***	773.9456	1529.8574	765.4323	1528.8734	764.9403	V	***375.2602***	188.1337	358.2336	179.6205	357.2496	179.1285	3
16	1633.9159	817.4616	1616.8894	808.9483	1615.9054	808.4563	S	276.1918	138.5995	259.1652	130.0863	258.1812	129.5942	2
17							K	189.1598	95.0835	172.1332	86.5702			1

RBCL, AtCg00490; K32

LTYYTPEYET**K**$_{32}$DTDILAAFR, expmz 2451.205902

Interpreted with: K11, Trimethyl (K)

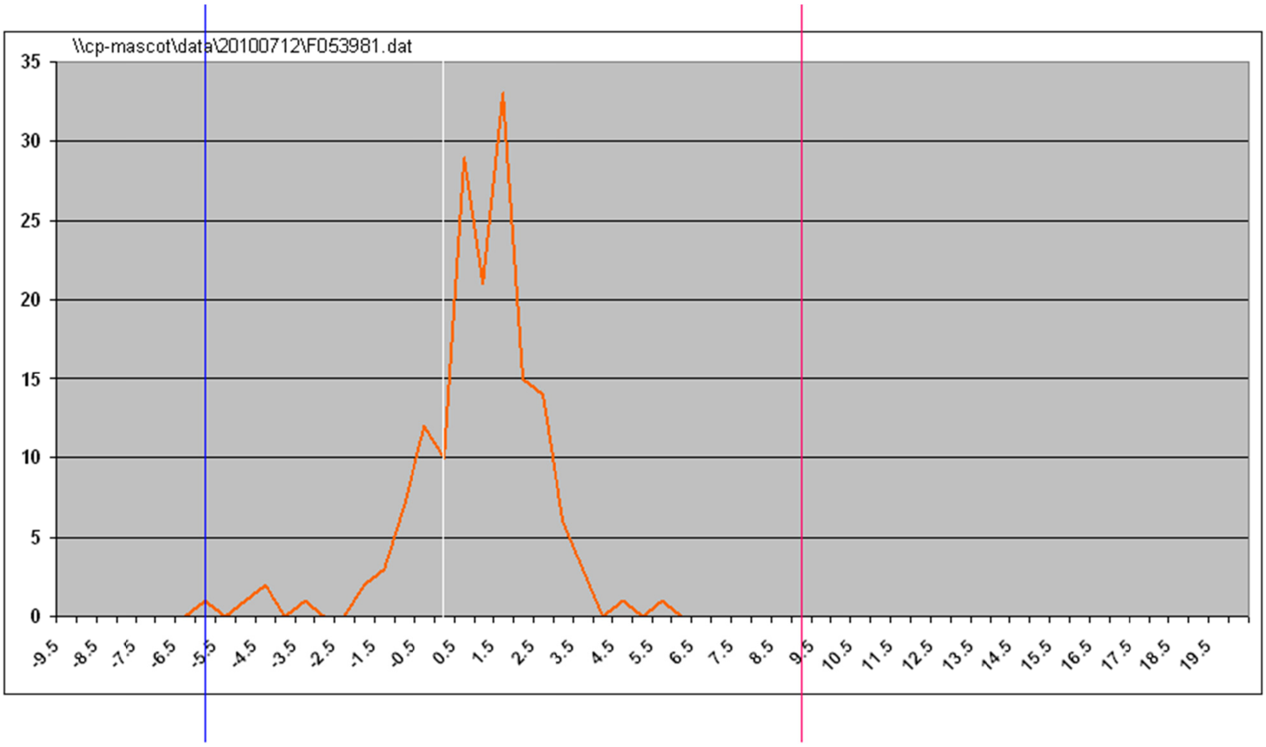

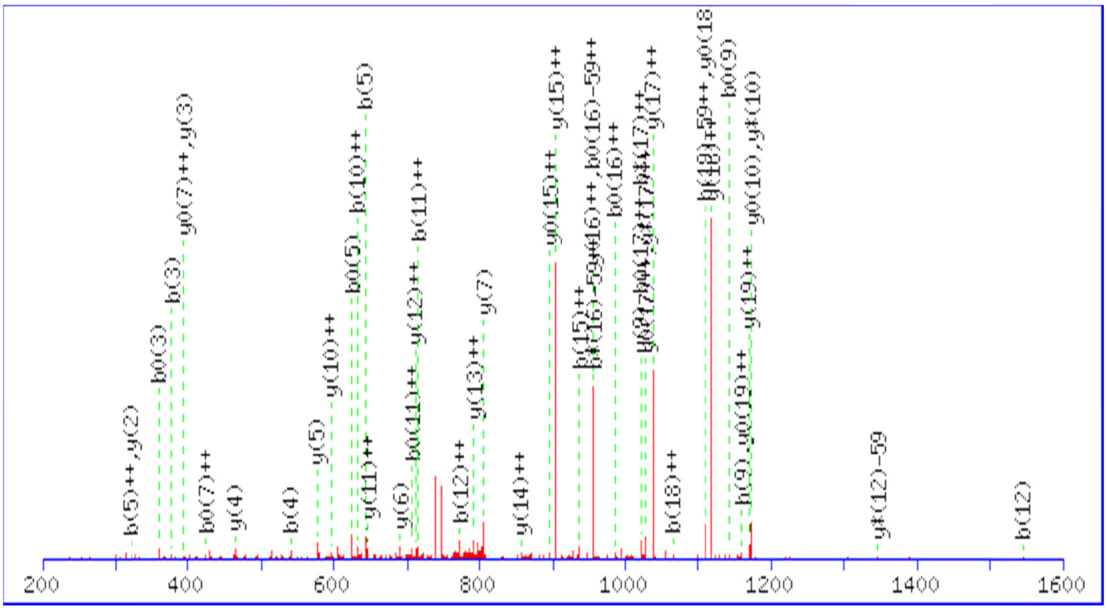

Zoom in the 800-1200 ppm range:

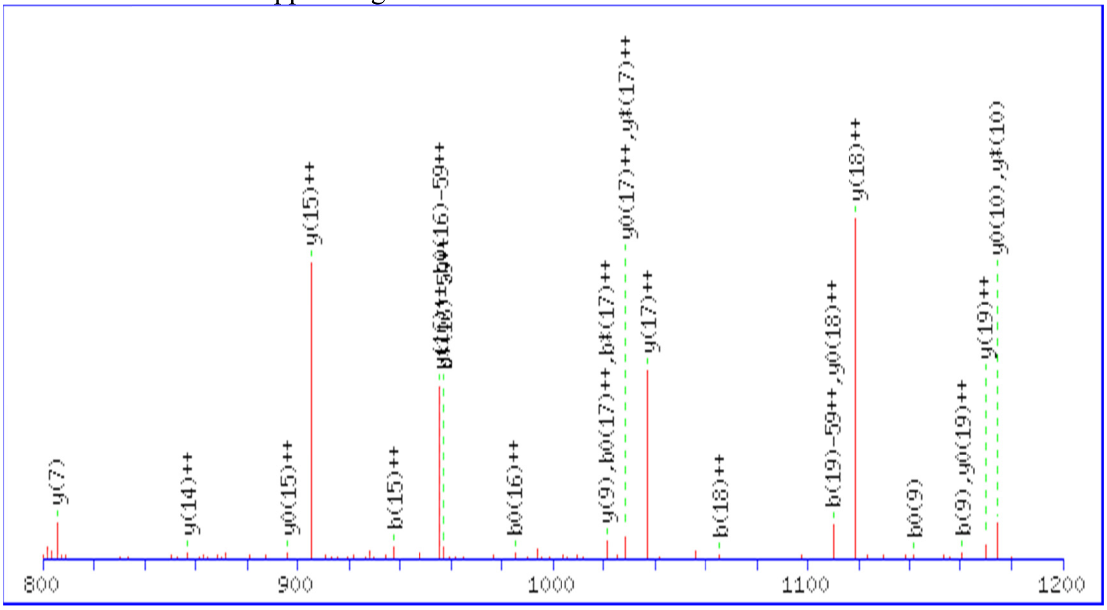

#	b	b⁺⁺	b*	b*⁺⁺	b⁰	b⁰⁺⁺	Seq.	y	y⁺⁺	y*	y*⁺⁺	y⁰	y⁰⁺⁺	#
1	114.0913	57.5493					L							20
2	215.1390	108.0731			197.1285	99.0679	T	2339.1442	1170.0757	2322.1176	1161.5625	2321.1336	1161.0704	19
3	**378.2023**	189.6048			360.1918	180.5995	Y	2238.0965	1119.5519	2221.0699	1111.0386	2220.0859	1110.5466	18
4	**541.2657**	271.1365			523.2551	262.1312	Y	2075.0332	1038.0202	2058.0066	1029.5069	2057.0226	1029.0149	17
5	**642.3134**	**321.6603**			624.3028	312.6550	T	1911.9698	956.4886	1894.9433	947.9753	1893.9593	947.4833	16
6	739.3661	370.1867			721.3556	361.1814	P	1810.9222	905.9647	1793.8956	897.4514	1792.9116	896.9594	15
7	868.4087	434.7080			850.3981	425.7027	E	1713.8694	857.4383	1696.8428	848.9251	1695.8588	848.4331	14
8	1031.4720	516.2397			1013.4615	507.2344	Y	1584.8268	792.9170	1567.8003	784.4038	1566.8162	783.9118	13
9	**1160.5146**	580.7610			1142.5041	571.7557	E	1421.7635	711.3854	1404.7369	702.8721	1403.7529	702.3801	12
10	1261.5623	**631.2848**			1243.5517	622.2795	T	1292.7209	646.8641	1275.6943	638.3508	1274.7103	637.8588	11
11	1431.7042	**716.3558**	1414.6777	707.8425	1413.6937	707.3505	K	1191.6732	596.3402	1174.6467	587.8270	1173.6626	587.3350	10
12	**1546.7312**	**773.8692**	1529.7046	765.3559	1528.7206	764.8639	D	***1021.5313***	511.2693	1004.5047	502.7560	1003.5207	502.2640	9
13	1647.7788	824.3931	1630.7523	815.8798	1629.7683	815.3878	T	906.5043	453.7558	889.4778	445.2425	888.4938	444.7505	8
14	1762.8058	881.9065	1745.7792	873.3933	1744.7952	872.9013	D	***805.4567***	403.2320	788.4301	394.7187	787.4461	394.2267	7
15	1875.8899	**938.4486**	1858.8633	929.9353	1857.8793	929.4433	I	***690.4297***	345.7185	673.4032	337.2052			6
16	1988.9739	994.9906	1971.9474	986.4773	1970.9634	985.9853	L	***577.3457***	289.1765	560.3191	280.6632			5
17	2060.0110	1030.5092	2042.9845	1021.9959	2042.0005	1021.5039	A	***464.2616***	232.6344	447.2350	224.1212			4
18	2131.0481	**1066.0277**	2114.0216	1057.5144	2113.0376	1057.0224	A	***393.2245***	197.1159	376.1979	188.6026			3
19	2278.1166	1139.5619	2261.0900	1131.0486	2260.1060	1130.5566	F	***322.1874***	161.5973	305.1608	153.0840			2
20							R	175.1190	88.0631	158.0924	79.5498			1

RBCL, AtCg00490; K236

SQAETGEI**K**$_{236}$GHYLNATAGTCEEMIK, expmz 2802.254142

Interpreted with: K9, Trimethyl (K); M23, Dioxidation (M)

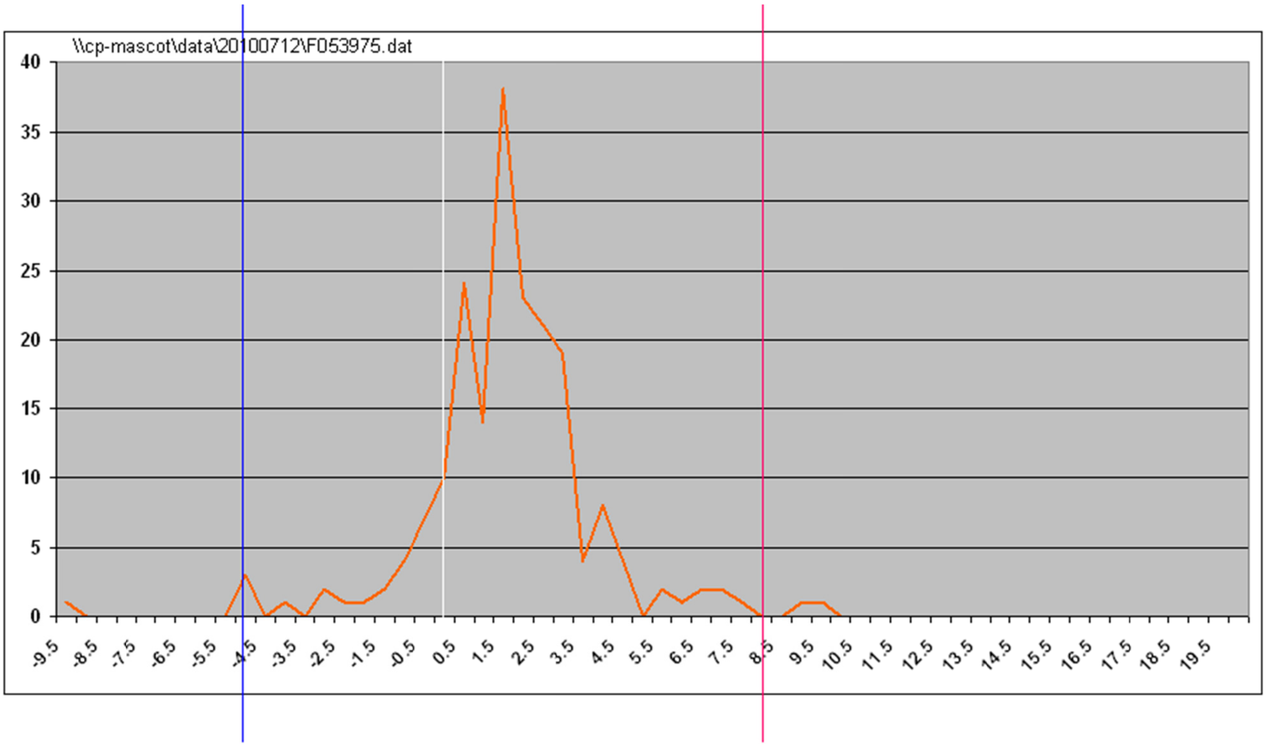

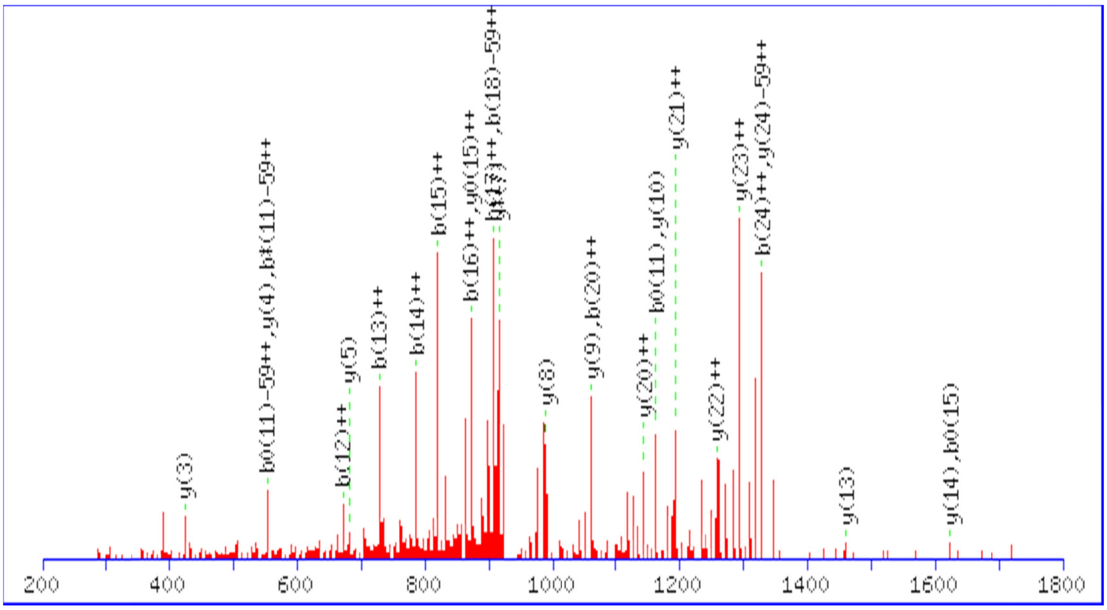

Zoom in the 500-1400 ppm range:

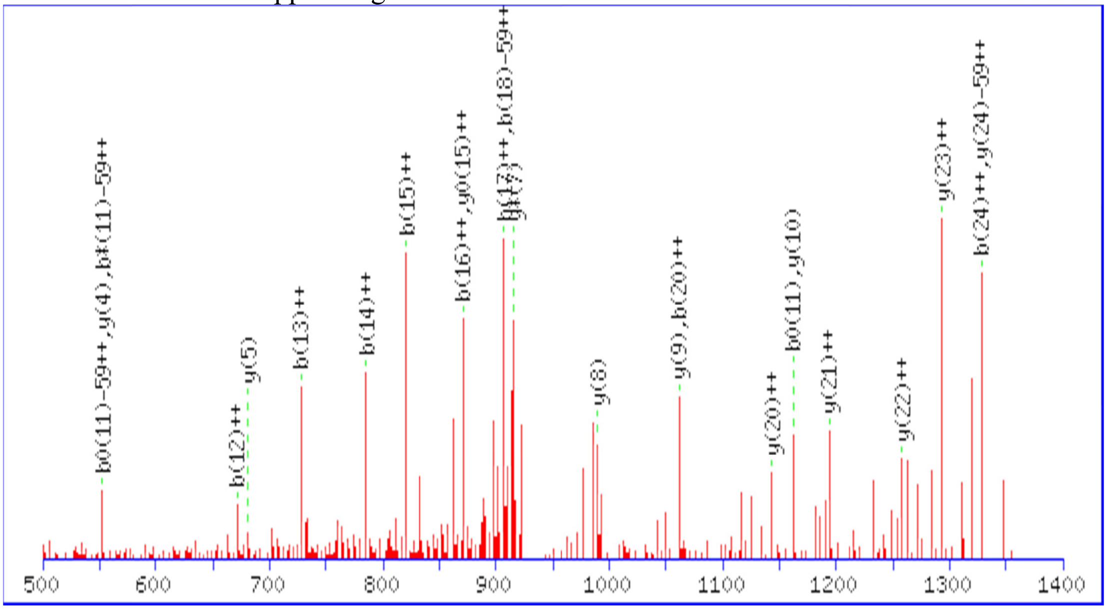

#	b	b^{++}	b*	b*$^{++}$	b^{0}	b^{0++}	Seq.	y	y^{++}	y*	y*$^{++}$	y^{0}	y^{0++}	#
1	88.0393	44.5233			70.0287	35.5180	S							25
2	216.0979	108.5526	199.0713	100.0393	198.0873	99.5473	Q	2716.2440	1358.6257	2699.2175	1350.1124	2698.2335	1349.6204	24
3	287.1350	144.0711	270.1084	135.5579	269.1244	135.0659	A	2588.1855	1294.5964	2571.1589	1286.0831	2570.1749	1285.5911	23
4	416.1776	208.5924	399.1510	200.0792	398.1670	199.5872	E	2517.1483	1259.0778	2500.1218	1250.5645	2499.1378	1250.0725	22
5	517.2253	259.1163	500.1987	250.6030	499.2147	250.1110	T	2388.1058	1194.5565	2371.0792	1186.0432	2370.0952	1185.5512	21
6	574.2467	287.6270	557.2202	279.1137	556.2362	278.6217	G	2287.0581	1144.0327	2270.0315	1135.5194	2269.0475	1135.0274	20
7	703.2893	352.1483	686.2628	343.6350	685.2788	343.1430	E	2230.0366	1115.5219	2213.0101	1107.0087	2212.0260	1106.5167	19
8	816.3734	408.6903	799.3468	400.1771	798.3628	399.6851	I	2100.9940	1051.0006	2083.9675	1042.4874	2082.9835	1041.9954	18
9	986.5153	493.7613	969.4888	485.2480	968.5047	484.7560	K	1987.9100	994.4586	1970.8834	985.9453	1969.8994	985.4533	17
10	1043.5368	522.2720	1026.5102	513.7587	1025.5262	513.2667	G	1817.7680	909.3877	1800.7415	900.8744	1799.7575	900.3824	16
11	1180.5957	590.8015	1163.5691	582.2882	1162.5851	581.7962	H	1760.7466	880.8769	1743.7200	872.3637	1742.7360	871.8716	15
12	1343.6590	***672.3331***	1326.6325	663.8199	1325.6484	663.3279	Y	***1623.6877***	812.3475	1606.6611	803.8342	1605.6771	803.3422	14
13	1456.7431	***728.8752***	1439.7165	720.3619	1438.7325	719.8699	L	***1460.6243***	730.8158	1443.5978	722.3025	1442.6138	721.8105	13
14	1570.7860	***785.8966***	1553.7594	777.3834	1552.7754	776.8914	N	1347.5403	674.2738	1330.5137	665.7605	1329.5297	665.2685	12
15	1641.8231	***821.4152***	1624.7966	812.9019	1623.8125	812.4099	A	1233.4973	617.2523	1216.4708	608.7390	1215.4868	608.2470	11
16	1742.8708	***871.9390***	1725.8442	863.4258	1724.8602	862.9338	T	***1162.4602***	581.7338	1145.4337	573.2205	1144.4497	572.7285	10
17	1813.9079	***907.4576***	1796.8814	898.9443	1795.8973	898.4523	A	***1061.4126***	531.2099	1044.3860	522.6966	1043.4020	522.2046	9
18	1870.9294	935.9683	1853.9028	927.4550	1852.9188	926.9630	G	***990.3754***	495.6914	973.3489	487.1781	972.3649	486.6861	8
19	1971.9770	986.4922	1954.9505	977.9789	1953.9665	977.4869	T	933.3540	467.1806	916.3274	458.6674	915.3434	458.1753	7
20	2122.9710	***1061.9891***	2105.9444	1053.4759	2104.9604	1052.9838	C	832.3063	416.6568	815.2797	408.1435	814.2957	407.6515	6
21	2252.0136	1126.5104	2234.9870	1117.9971	2234.0030	1117.5051	E	***681.3124***	341.1598	664.2858	332.6465	663.3018	332.1545	5
22	2381.0562	1191.0317	2364.0296	1182.5184	2363.0456	1182.0264	E	***552.2698***	276.6385	535.2432	268.1253	534.2592	267.6332	4
23	2544.0865	1272.5469	2527.0599	1264.0336	2526.0759	1263.5416	M	***423.2272***	212.1172	406.2006	203.6040			3
24	2657.1705	***1329.0889***	2640.1440	1320.5756	2639.1600	1320.0836	I	260.1969	130.6021	243.1703	122.0888			2
25							K	147.1128	74.0600	130.0863	65.5468			1

TIC62, AT3G18890.1

ASSVVTEASPTNLNS**K**$_{79}$EEDLVFVAGATGK, mzexp 2962.489962

Interpreted with: K16, Trimethyl (K)

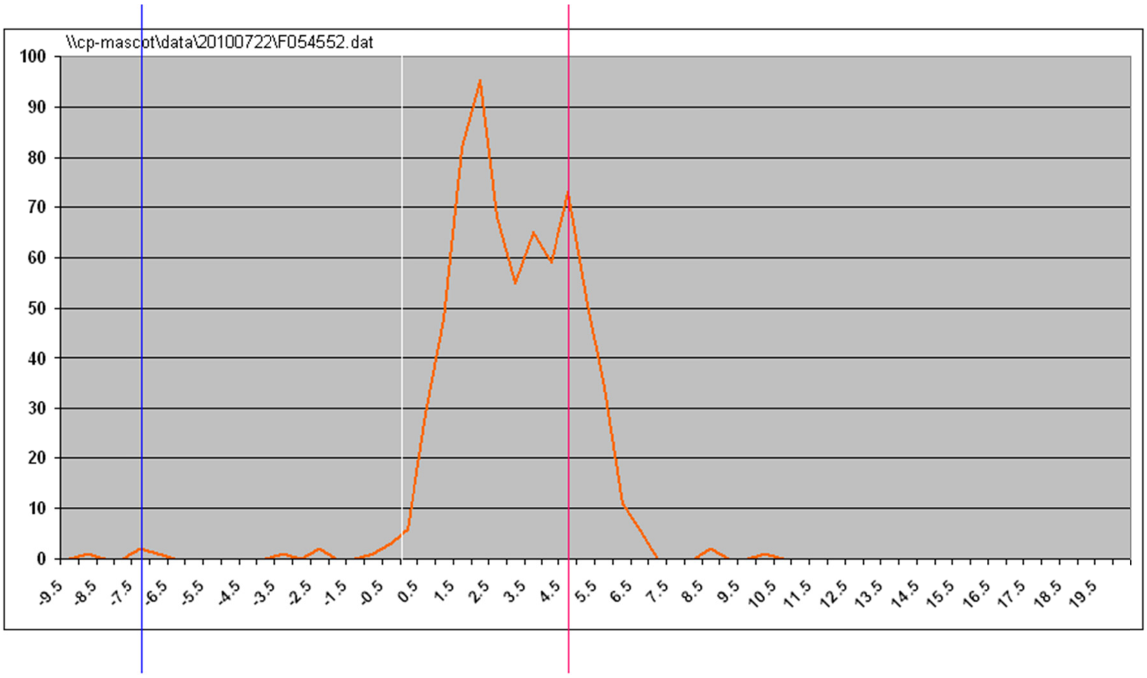

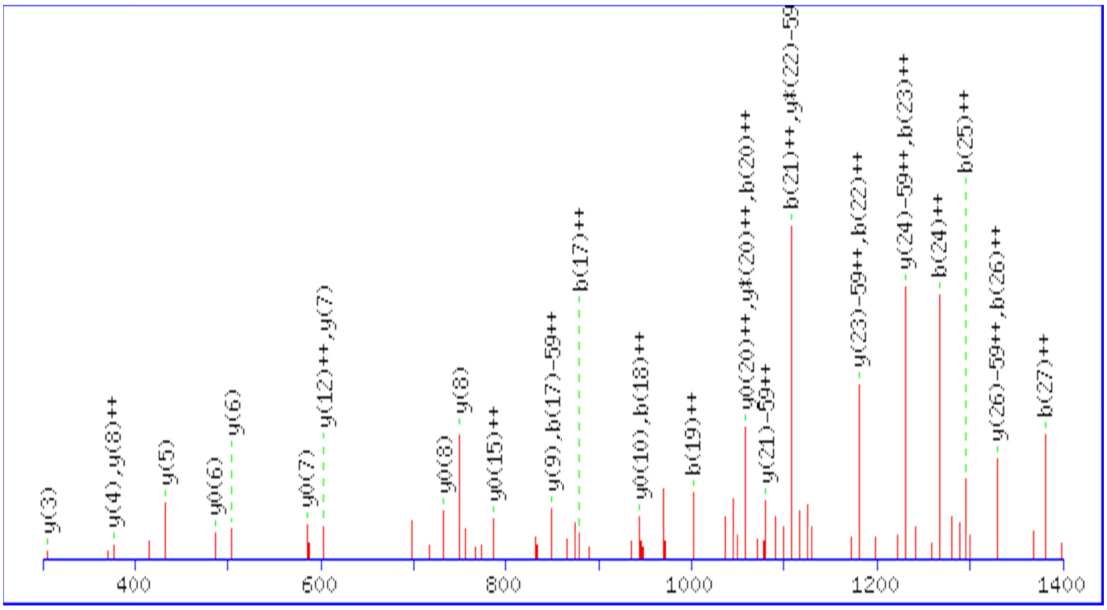

#	b	b^{++}	b*	b^{*++}	b^{0}	b^{0++}	Seq.	y	y^{++}	y*	y^{*++}	y^{0}	y^{0++}	#
1	72.0444	36.5258					A							29
2	159.0764	80.0418			141.0659	71.0366	S	2892.4837	1446.7455	2875.4571	1438.2322	2874.4731	1437.7402	28
3	246.1084	123.5579			228.0979	114.5526	S	2805.4516	1403.2295	2788.4251	1394.7162	2787.4411	1394.2242	27
4	345.1769	173.0921			327.1663	164.0868	V	2718.4196	1359.7134	2701.3931	1351.2002	2700.4091	1350.7082	26
5	444.2453	222.6263			426.2347	213.6210	V	2619.3512	1310.1792	2602.3247	1301.6660	2601.3406	1301.1740	25
6	545.2930	273.1501			527.2824	264.1448	T	2520.2828	1260.6450	2503.2562	1252.1318	2502.2722	1251.6398	24
7	674.3355	337.6714			656.3250	328.6661	E	2419.2351	1210.1212	2402.2086	1201.6079	2401.2245	1201.1159	23
8	745.3727	373.1900			727.3621	364.1847	A	2290.1925	1145.5999	2273.1660	1137.0866	2272.1820	1136.5946	22
9	832.4047	416.7060			814.3941	407.7007	S	2219.1554	1110.0813	2202.1289	1101.5681	2201.1448	1101.0761	21
10	929.4575	465.2324			911.4469	456.2271	P	2132.1234	1066.5653	2115.0968	1058.0521	2114.1128	1057.5600	20
11	1030.5051	515.7562			1012.4946	506.7509	T	2035.0706	1018.0389	2018.0441	1009.5257	2017.0600	1009.0337	19
12	1144.5481	572.7777	1127.5215	564.2644	1126.5375	563.7724	N	1934.0229	967.5151	1916.9964	959.0018	1916.0124	958.5098	18
13	1257.6321	629.3197	1240.6056	620.8064	1239.6216	620.3144	L	1819.9800	910.4936	1802.9535	901.9804	1801.9694	901.4884	17
14	1371.6750	686.3412	1354.6485	677.8279	1353.6645	677.3359	N	1706.8959	853.9516	1689.8694	845.4383	1688.8854	844.9463	16
15	1458.7071	729.8572	1441.6805	721.3439	1440.6965	720.8519	S	1592.8530	796.9301	1575.8265	788.4169	1574.8424	787.9249	15
16	1628.8490	814.9281	1611.8224	806.4149	1610.8384	805.9229	K	1505.8210	753.4141	1488.7944	744.9009	1487.8104	744.4088	14
17	1757.8916	***879.4494***	1740.8650	870.9362	1739.8810	870.4441	E	1335.6791	668.3432	1318.6525	659.8299	1317.6685	659.3379	13
18	1886.9342	***943.9707***	1869.9076	935.4575	1868.9236	934.9654	E	1206.6365	603.8219	1189.6099	595.3086	1188.6259	594.8166	12
19	2001.9611	***1001.4842***	1984.9346	992.9709	1983.9506	992.4789	D	1077.5939	539.3006	1060.5673	530.7873	1059.5833	530.2953	11
20	2115.0452	***1058.0262***	2098.0186	1049.5130	2097.0346	1049.0209	L	962.5669	481.7871	945.5404	473.2738	944.5564	472.7818	10
21	2214.1136	***1107.5604***	2197.0870	1099.0472	2196.1030	1098.5552	V	**849.4829**	425.2451	832.4563	416.7318	831.4723	416.2398	9
22	2361.1820	***1181.0946***	2344.1555	1172.5814	2343.1714	1172.0894	F	**750.4145**	375.7109	733.3879	367.1976	732.4039	366.7056	8
23	2460.2504	***1230.6289***	2443.2239	1222.1156	2442.2399	1221.6236	V	**603.3461**	302.1767	586.3195	293.6634	585.3355	293.1714	7
24	2531.2875	***1266.1474***	2514.2610	1257.6341	2513.2770	1257.1421	A	**504.2776**	252.6425	487.2511	244.1292	486.2671	243.6372	6
25	2588.3090	***1294.6581***	2571.2825	1286.1449	2570.2984	1285.6529	G	**433.2405**	217.1239	416.2140	208.6106	415.2300	208.1186	5
26	2659.3461	***1330.1767***	2642.3196	1321.6634	2641.3356	1321.1714	A	**376.2191**	188.6132	359.1925	180.0999	358.2085	179.6079	4
27	2760.3938	***1380.7005***	2743.3672	1372.1873	2742.3832	1371.6953	T	**305.1819**	153.0946	288.1554	144.5813	287.1714	144.0893	3
28	2817.4153	1409.2113	2800.3887	1400.6980	2799.4047	1400.2060	G	204.1343	102.5708	187.1077	94.0575			2
29							K	147.1128	74.0600	130.0863	65.5468			1

SDH, AT5G39410.1

MNPTQ**K**$_{\mathbf{6}}$PEPVYDMVILGASGFTGK, expmz 2621.318802

Interpreted with: K6, Trimethyl (K)

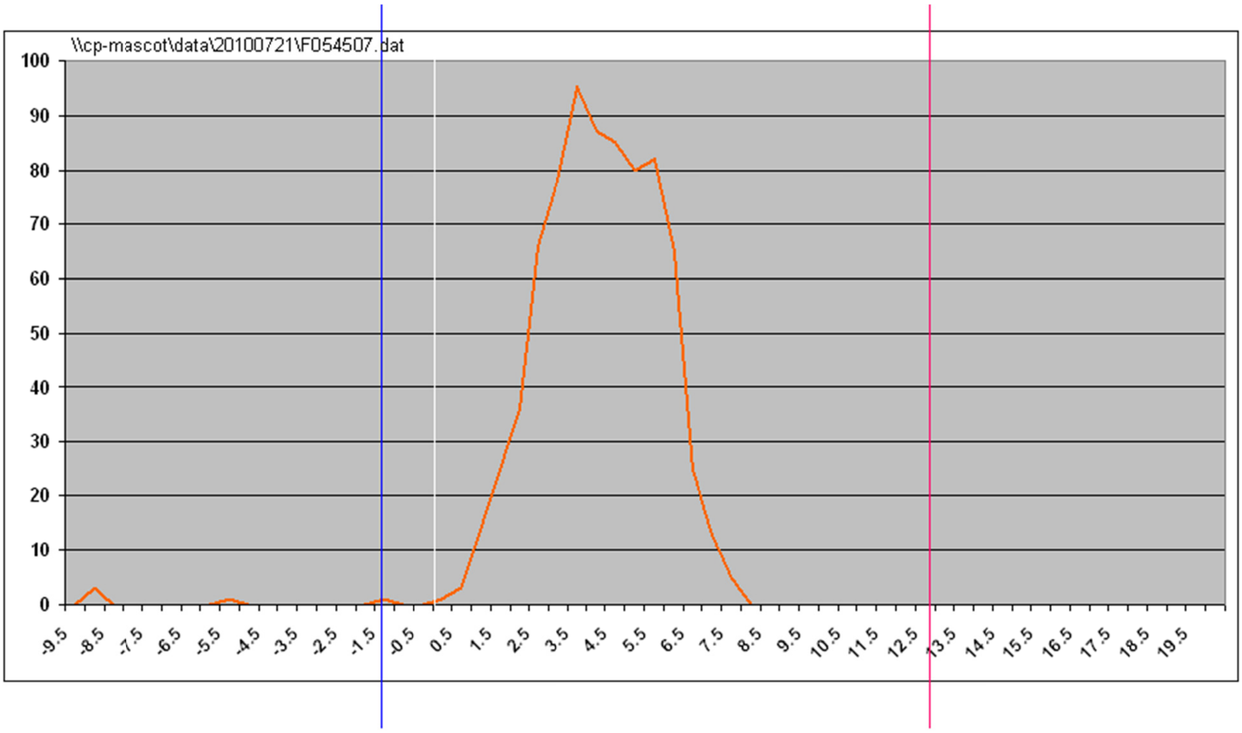

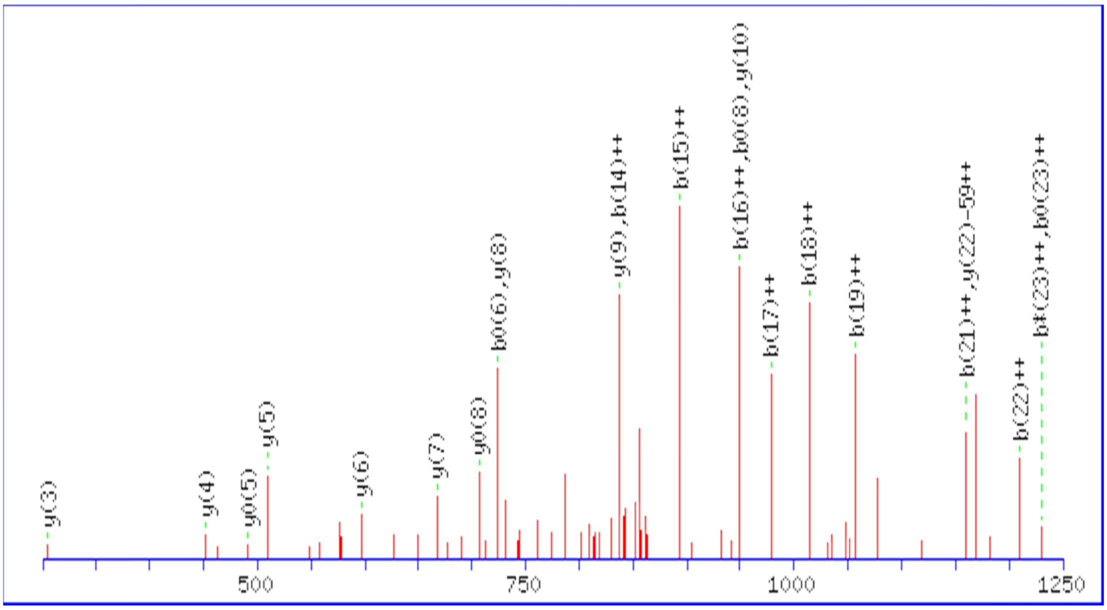

#	b	b^{++}	b*	b^{*++}	b^{0}	b^{0++}	Seq.	y	y^{++}	y*	y^{*++}	y^{0}	y^{0++}	#
1	132.0478	66.5275					M							24
2	246.0907	123.5490	229.0641	115.0357			N	2491.2901	1246.1487	2474.2636	1237.6354	2473.2796	1237.1434	23
3	343.1435	172.0754	326.1169	163.5621			P	2377.2472	1189.1272	2360.2206	1180.6140	2359.2366	1180.1220	22
4	444.1911	222.5992	427.1646	214.0859	426.1806	213.5939	T	2280.1944	1140.6009	2263.1679	1132.0876	2262.1839	1131.5956	21
5	572.2497	286.6285	555.2232	278.1152	554.2391	277.6232	Q	2179.1468	1090.0770	2162.1202	1081.5637	2161.1362	1081.0717	20
6	742.3916	371.6994	725.3651	363.1862	724.3811	362.6942	K	2051.0882	1026.0477	2034.0616	1017.5345	2033.0776	1017.0424	19
7	839.4444	420.2258	822.4178	411.7126	821.4338	411.2205	P	1880.9463	940.9768	1863.9197	932.4635	1862.9357	931.9715	18
8	968.4870	484.7471	951.4604	476.2339	950.4764	475.7418	E	1783.8935	892.4504	1766.8670	883.9371	1765.8829	883.4451	17
9	1065.5397	533.2735	1048.5132	524.7602	1047.5292	524.2682	P	1654.8509	827.9291	1637.8244	819.4158	1636.8403	818.9238	16
10	1164.6082	582.8077	1147.5816	574.2944	1146.5976	573.8024	V	1557.7981	779.4027	1540.7716	770.8894	1539.7876	770.3974	15
11	1327.6715	664.3394	1310.6449	655.8261	1309.6609	655.3341	Y	1458.7297	729.8685	1441.7032	721.3552	1440.7192	720.8632	14
12	1442.6984	721.8529	1425.6719	713.3396	1424.6879	712.8476	D	1295.6664	648.3368	1278.6399	639.8236	1277.6558	639.3316	13
13	1573.7389	787.3731	1556.7124	778.8598	1555.7283	778.3678	M	1180.6395	590.8234	1163.6129	582.3101	1162.6289	581.8181	12
14	1672.8073	***836.9073***	1655.7808	828.3940	1654.7968	827.9020	V	1049.5990	525.3031	1032.5724	516.7898	1031.5884	516.2978	11
15	1785.8914	***893.4493***	1768.8648	884.9361	1767.8808	884.4441	I	***950.5306***	475.7689	933.5040	467.2556	932.5200	466.7636	10
16	1898.9755	***949.9914***	1881.9489	941.4781	1880.9649	940.9861	L	***837.4465***	419.2269	820.4199	410.7136	819.4359	410.2216	9
17	1955.9969	***978.5021***	1938.9704	969.9888	1937.9864	969.4968	G	***724.3624***	362.6849	707.3359	354.1716	706.3519	353.6796	8
18	2027.0340	***1014.0207***	2010.0075	1005.5074	2009.0235	1005.0154	A	***667.3410***	334.1741	650.3144	325.6608	649.3304	325.1688	7
19	2114.0661	***1057.5367***	2097.0395	1049.0234	2096.0555	1048.5314	S	***596.3039***	298.6556	579.2773	290.1423	578.2933	289.6503	6
20	2171.0875	1086.0474	2154.0610	1077.5341	2153.0770	1077.0421	G	***509.2718***	255.1396	492.2453	246.6263	491.2613	246.1343	5
21	2318.1559	***1159.5816***	2301.1294	1151.0683	2300.1454	1150.5763	F	***452.2504***	226.6288	435.2238	218.1155	434.2398	217.6235	4
22	2419.2036	***1210.1054***	2402.1771	1201.5922	2401.1931	1201.1002	T	***305.1819***	153.0946	288.1554	144.5813	287.1714	144.0893	3
23	2476.2251	1238.6162	2459.1985	1230.1029	2458.2145	1229.6109	G	204.1343	102.5708	187.1077	94.0575			2
24							K	147.1128	74.0600	130.0863	65.5468			1

PRPL11, AT1G32990.1

GVNIMAFC**K**$_{109}$DYNAR, expmz 1722.784188

Interpreted with: M5, Dioxidation (M); K9, Trimethyl (K)

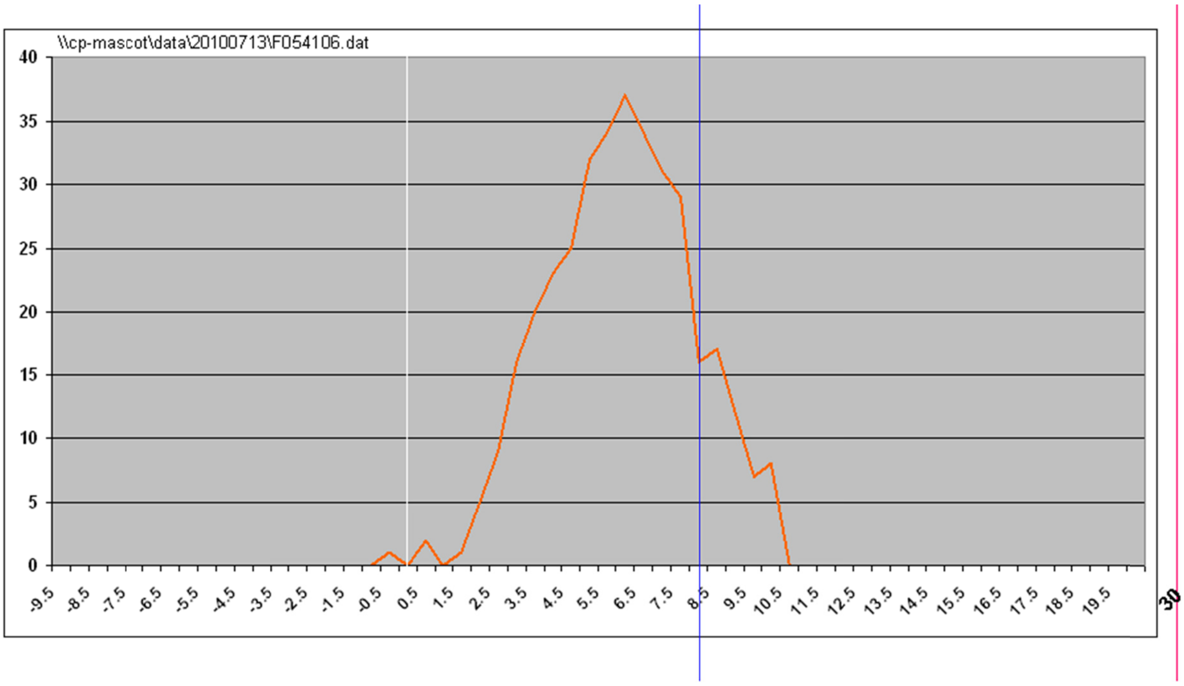

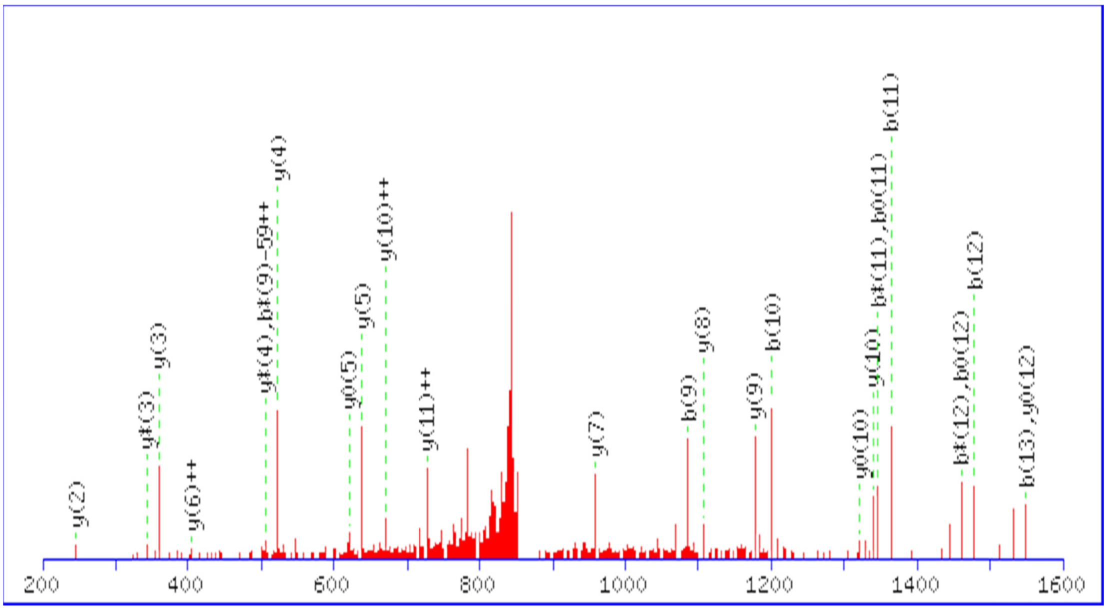

#	b	b^{++}	b*	b^{*++}	b^{0}	b^{0++}	Seq.	y	y^{++}	y*	y^{*++}	y^{0}	y^{0++}	#
1	58.0287	29.5180					G							14
2	157.0972	79.0522					V	1666.7564	833.8818	1649.7298	825.3685	1648.7458	824.8765	13
3	271.1401	136.0737	254.1135	127.5604			N	1567.6879	784.3476	1550.6614	775.8343	1549.6774	775.3423	12
4	384.2241	192.6157	367.1976	184.1024			I	1453.6450	727.3261	1436.6185	718.8129	1435.6344	718.3209	11
5	547.2545	274.1309	530.2279	265.6176			M	***1340.5609***	670.7841	1323.5344	662.2708	1322.5504	661.7788	10
6	618.2916	309.6494	601.2650	301.1362			A	***1177.5306***	589.2690	1160.5041	580.7557	1159.5201	580.2637	9
7	765.3600	383.1836	748.3334	374.6704			F	***1106.4935***	553.7504	1089.4670	545.2371	1088.4830	544.7451	8
8	916.3539	458.6806	899.3274	450.1673			C	***959.4251***	480.2162	942.3986	471.7029	941.4145	471.2109	7
9	**1086.4958**	543.7516	1069.4693	535.2383			K	808.4312	404.7192	791.4046	396.2060	790.4206	395.7139	6
10	**1201.5228**	601.2650	1184.4962	592.7517	1183.5122	592.2597	D	***638.2893***	319.6483	621.2627	311.1350	620.2787	310.6430	5
11	**1364.5861**	682.7967	1347.5596	674.2834	1346.5755	673.7914	Y	***523.2623***	262.1348	506.2358	253.6215			4
12	**1478.6290**	739.8182	1461.6025	731.3049	1460.6185	730.8129	N	***360.1990***	180.6031	343.1724	172.0899			3
13	**1549.6661**	775.3367	1532.6396	766.8234	1531.6556	766.3314	A	***246.1561***	123.5817	229.1295	115.0684			2
14							R	175.1190	88.0631	158.0924	79.5498			1

AT2G33090.1

SK_{79}TGEVTREK, expmz 1175.657808

Interpreted with: K2, Trimethyl (K)

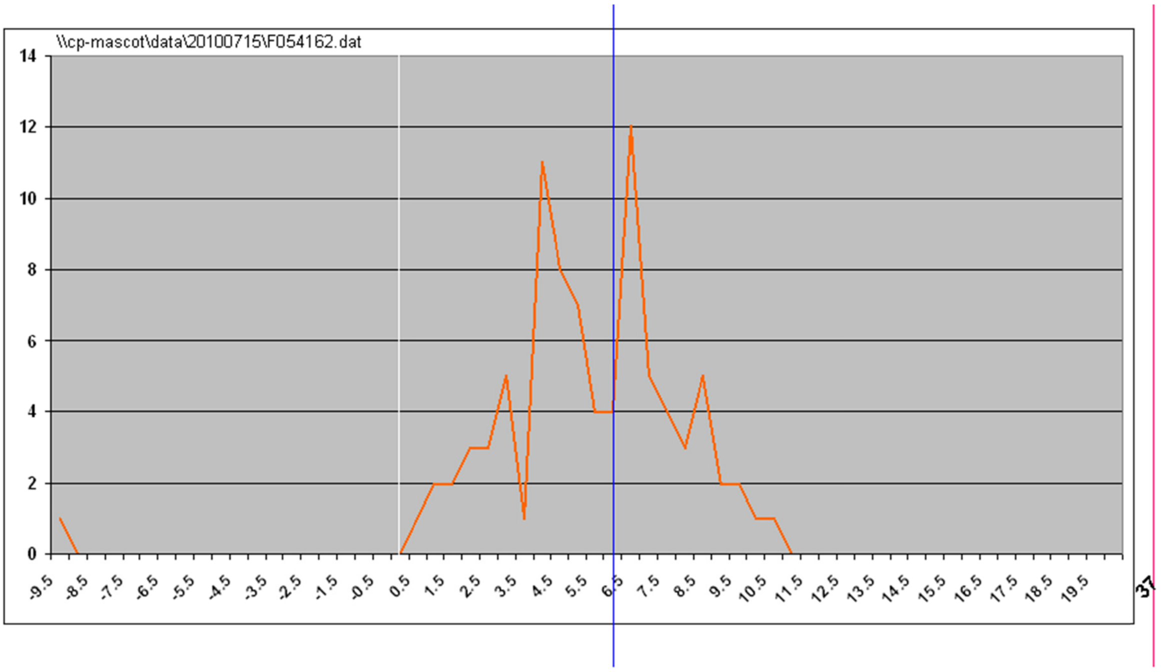

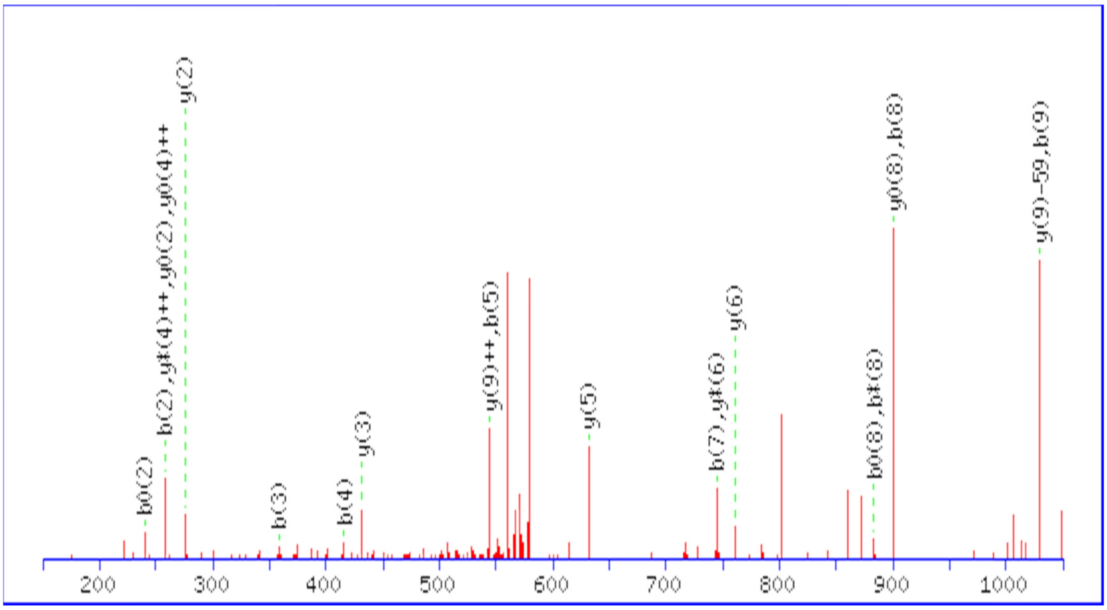

#	b	b^{++}	b*	b^{*++}	b^{0}	b^{0++}	Seq.	y	y^{++}	y*	y^{*++}	y^{0}	y^{0++}	#
1	88.0393	44.5233			70.0287	35.5180	S							10
2	***258.1812***	129.5942	241.1547	121.0810	240.1707	120.5890	K	1089.6263	545.3168	1072.5997	536.8035	1071.6157	536.3115	9
3	***359.2289***	180.1181	342.2023	171.6048	341.2183	171.1128	T	919.4843	460.2458	902.4578	451.7325	901.4738	451.2405	8
4	***416.2504***	208.6288	399.2238	200.1155	398.2398	199.6235	G	818.4367	409.7220	801.4101	401.2087	800.4261	400.7167	7
5	***545.2930***	273.1501	528.2664	264.6368	527.2824	264.1448	E	**761.4152**	381.2112	744.3886	372.6980	743.4046	372.2060	6
6	644.3614	322.6843	627.3348	314.1710	626.3508	313.6790	V	**632.3726**	316.6899	615.3461	308.1767	614.3620	307.6847	5
7	***745.4090***	373.2082	728.3825	364.6949	727.3985	364.2029	T	533.3042	267.1557	516.2776	258.6425	515.2936	258.1504	4
8	***901.5102***	451.2587	884.4836	442.7454	883.4996	442.2534	R	**432.2565**	216.6319	415.2300	208.1186	414.2459	207.6266	3
9	***1030.5528***	515.7800	1013.5262	507.2667	1012.5422	506.7747	E	**276.1554**	138.5813	259.1288	130.0681	258.1448	129.5761	2
10							K	147.1128	74.0600	130.0863	65.5468			1
