# Supplementary material for: Uncovering the Protein Lysine and Arginine Methylation Network in Arabidopsis Chloroplasts
Source: PLoS One. 2014 Apr 18;9(4):e95512. doi: 10.1371/journal.pone.0095512 (PMC3991674; doi:10.1371/journal.pone.0095512)

Supplemental Figure S2: Positioning of methylation sites on the 3D structure models of some identified methylproteins.

The 3D structures were modeled with Phyre2 server and imaged with PyMol software. In all cases, >60% (64-100%) residues were modeled with >90% (90-100%) confidence. ATP-synthase (alpha subunit: AtCg00120, beta subunit: ATCg00480) was fitted to ATP-synthase from spinach chloroplast (PDB entry 1FX0), fructose 1,6 bisphosphate aldolase (At2g21330 and At4g38970 isoforms) to aldolase from rabbit muscle (PDB entry 1ZAI), phosphoglycerate kinase (At3g12780) to phosphoglycerate kinase from *Bacillus stearothermophilus* (PDB entry 1PHP), protochlorophyllide reductase C (At1g03630) to salutaridine reductase from *Papaver somniferum* (PDB entry 3O26), inorganic pyrophosphatase 1 (At5g09650) to the yeast *Saccharomyces cerevisiae* inorganic pyrophosphatase (PDB entry 1E9G), ribose 5-phosphate isomerase (At3g04790) to ribose 5-phosphate isomerase A from *Burkholderia thailandensis* (PDB entry 3U7J), and 50S ribosomal protein L11 (At1g32990) to spinach chloroplast 50S subunit L11 (PDB entry 3BBO). Positions of methylated residues in Arabidopsis Rubisco (RbcS: At1g67090, RbcL: AtCg00490) are illustrated in the 3D structure of the spinach Rubisco (PDB entry 1RCX). Lys residues K11 and K91 in the spinach RbcS of Rubisco correspond to K66 and K146, respectively, in the Arabidopsis protein. Methylated residues are colored in yellow.

Fructose 1,6-bisphosphate aldolase
(FBA1: At2g21330, FBA2: At4g38970)

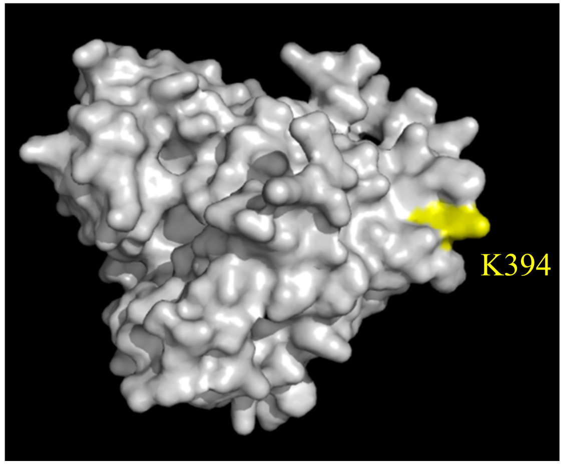

ATP synthase
(ATP-B: AtCg00480, dark grey;
ATP-A: AtCg00120, light grey)

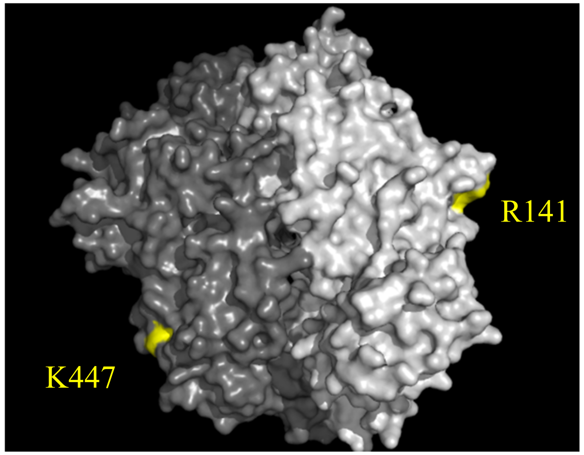

50S ribosomal protein L11
(At1g32990)

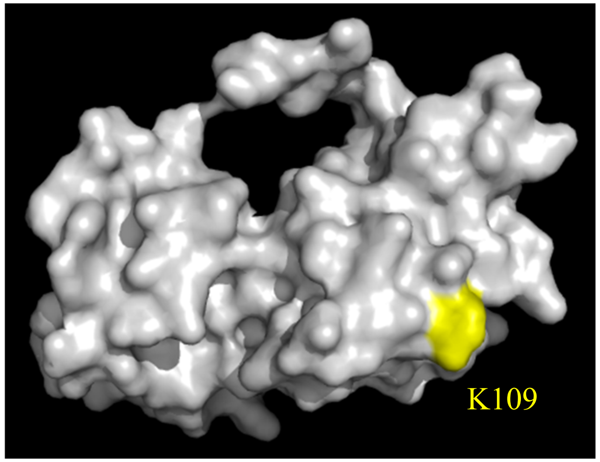

Phosphoglycerate kinase
(At3g12780)

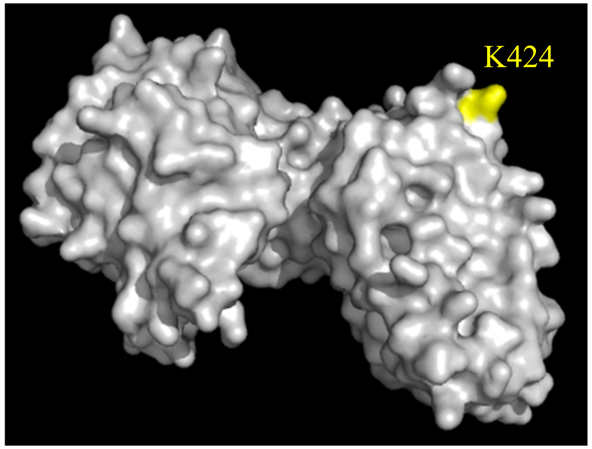

Protochlorophyllide reductase C
(At1g03630)

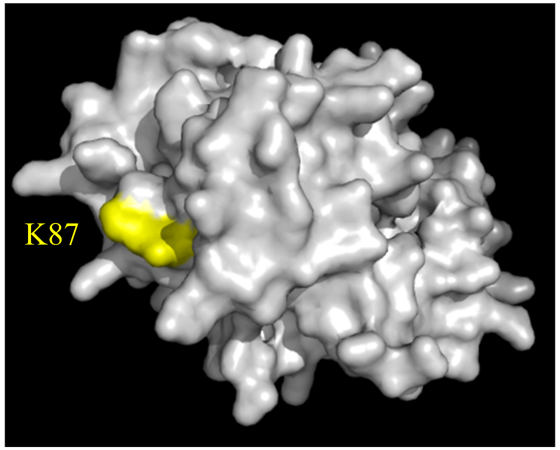

Inorganic pyrophosphatase 1
(At5g09650)

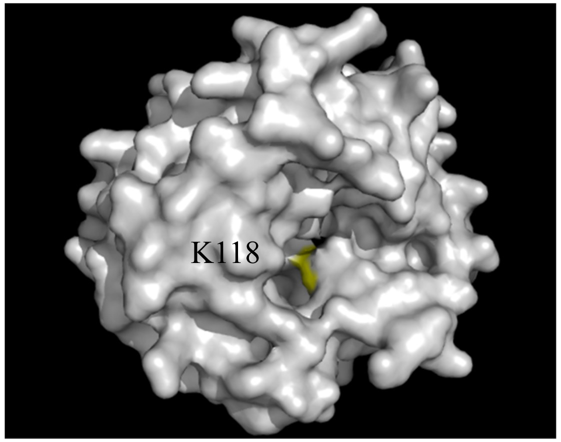

Ribose 5-phosphate isomerase
(At3g04790)

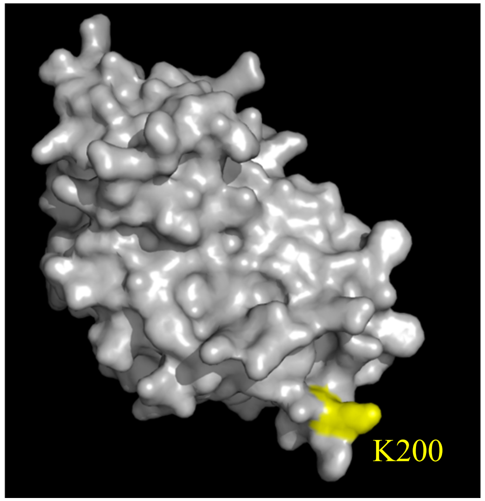

Rubisco (spinach enzyme; RbcS in light grey; RbcL in dark grey)

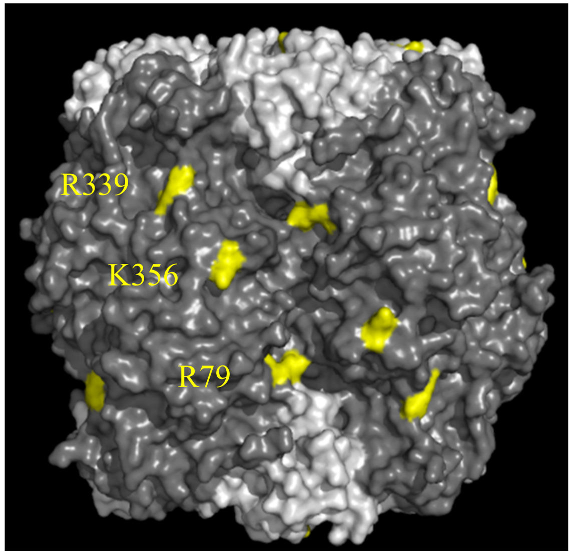

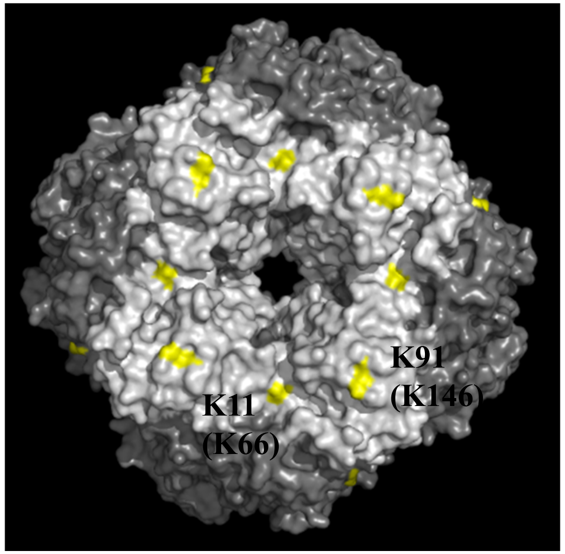

Supplement: Figure S2 — Positioning of methylation sites on the 3D structure models of some identified methylproteins. (PDF) [file pone.0095512.s002.pdf]
