# Supplementary material for: Uncovering the Protein Lysine and Arginine Methylation Network in Arabidopsis Chloroplasts
Source: PLoS One. 2014 Apr 18;9(4):e95512. doi: 10.1371/journal.pone.0095512 (PMC3991674; doi:10.1371/journal.pone.0095512)

Supplemental Figure S3: Documentation on recombinant protein substrates and methyltransferases used for *in vitro* methylation assays.

(a) Recombinant protein substrates (5-15 µg) were analyzed by SDS-PAGE. The molecular masses of purified proteins are: GAPA1, 38 kDa; ATP-B, 54 kDa; PRPL11, 18 kDa; FBA2, 40 kDa.
(b) Recombinant protein methyltransferases (2-4 µg) were analyzed by SDS-PAGE. The molecular masses of purified proteins are: LSMT-like, 51 kDa; PrmA-like, 34 kDa; PPKMT1, 51 kDa; PPKMT2, 52 kDa; PTAC14, 50 kDa.

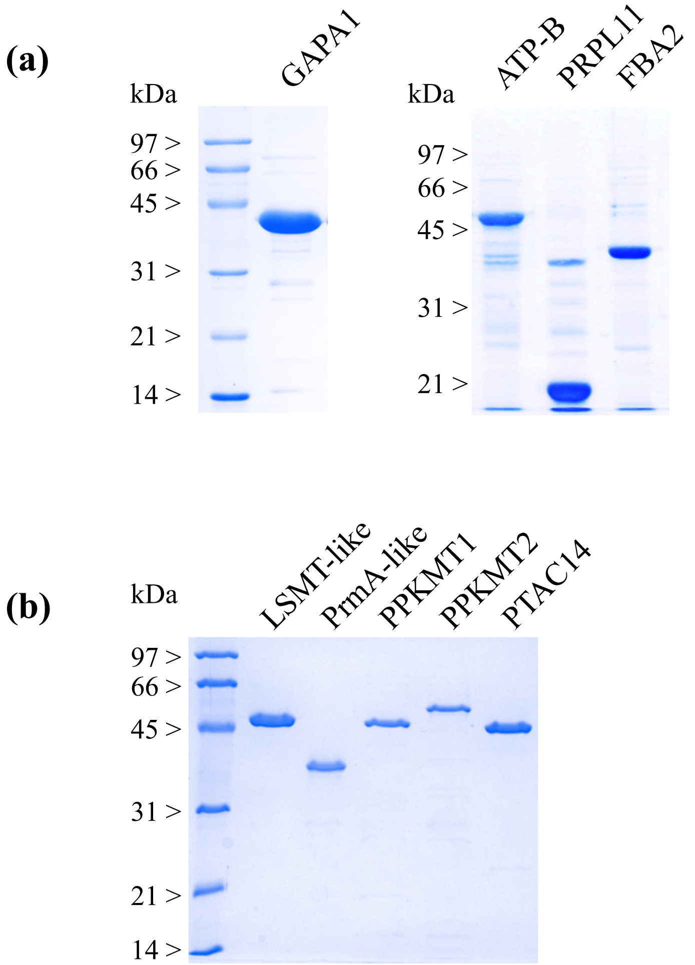

Supplement: Figure S3 — Documentation on recombinant protein substrates and methyltransferases used for in vitro methylation assays. (PDF) [file pone.0095512.s003.pdf]
