# Supplementary material for: Uncovering the Protein Lysine and Arginine Methylation Network in Arabidopsis Chloroplasts
Source: PLoS One. 2014 Apr 18;9(4):e95512. doi: 10.1371/journal.pone.0095512 (PMC3991674; doi:10.1371/journal.pone.0095512)

Supplemental Figure S4: LC-MS/MS fragmentation spectra of recombinant PRPL11 and ATP-B methylated *in vitro* by chloroplast stroma.
For PRPL11, spectra are from peptides bearing unmodified (control, no AdoMet added in the assay) or trimethylated (complete assay) Lys at position 109. For ATP-B, spectra are from peptides bearing unmodified (control, no AdoMet added in the assay) or dimethylated (complete assay) Lys at position 447.

PRPL11

- GVNIMAFCK$_{109}$DYNAR p. 2-3
- GVNIMAFCK$_{109(me3)}$DYNAR p. 4-5

ATP-B

- FLSQPFFVAEVFTGSPGK$_{447}$YVGLAETIR p. 6-7
- FLSQPFFVAEVFTGSPGK$_{447(me2)}$YVGLAETIR p. 8-9

MS/MS Fragmentation of **GVNIMAFCKDYNAR**
Found in **AT1G32990.1** in ATH_Cplet_D, | Symbols: **PRPL11** | plastid ribosomal protein l11 | chr1:11955827-11957139 FORWARD LENGTH=222

Match to Query 3014: 1657.771308 from(829.892930,2+) intensity(73143.0390) scans(4961) rawscans(sn4961) rtinseconds(1690.3875) index(4109)

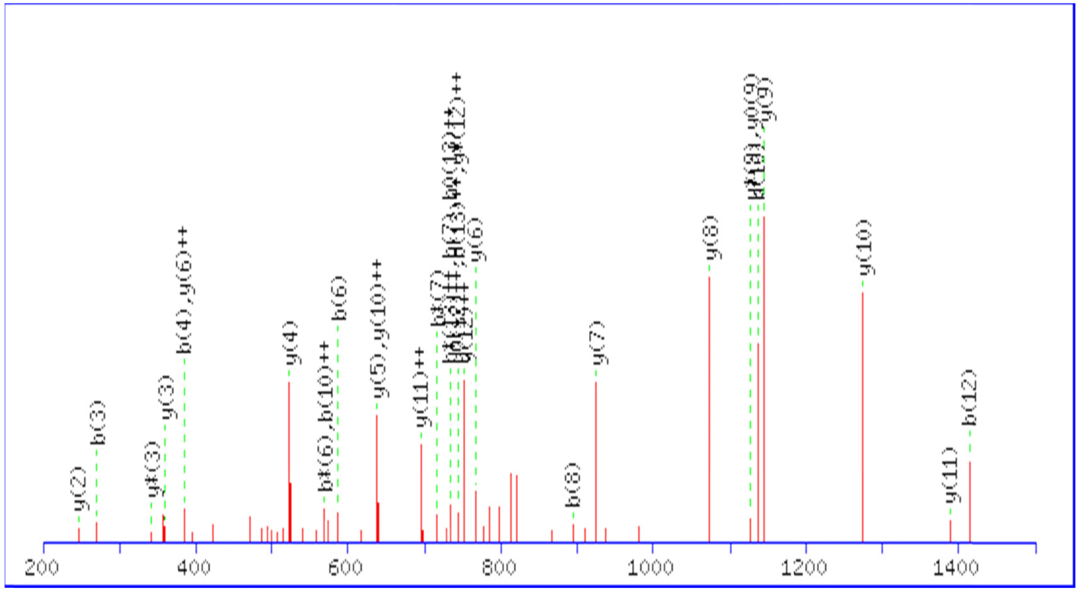

Monoisotopic mass of neutral peptide Mr(calc): 1657.7705
Fixed modifications: Carbamidomethyl (C) (apply to specified residues or termini only)
Ions Score: 85 **Expect:** 7.8e-07
Matches : 32/126 fragment ions using 35 most intense peaks

#	b	b^{++}	b*	b^{*++}	b^{0}	b^{0++}	Seq.	y	y^{++}	y*	y^{*++}	y^{0}	y^{0++}	#
1	58.0287	29.5180					G							14
2	157.0972	79.0522					V	1601.7563	801.3818	1584.7297	792.8685	1583.7457	792.3765	13
3	**271.1401**	136.0737	254.1135	127.5604			N	1502.6879	751.8476	1485.6613	743.3343	1484.6773	742.8423	12
4	**384.2241**	192.6157	367.1976	184.1024			I	***1388.6450***	694.8261	1371.6184	686.3128	1370.6344	685.8208	11
5	515.2646	258.1360	498.2381	249.6227			M	***1275.5609***	638.2841	1258.5343	629.7708	1257.5503	629.2788	10
6	**586.3017**	293.6545	569.2752	285.1412			A	***1144.5204***	572.7638	1127.4939	564.2506	1126.5098	563.7586	9
7	**733.3702**	367.1887	716.3436	358.6754			F	***1073.4833***	537.2453	1056.4567	528.7320	1055.4727	528.2400	8
8	**893.4008**	447.2040	876.3743	438.6908			C	***926.4149***	463.7111	909.3883	455.1978	908.4043	454.7058	7
9	1021.4958	511.2515	1004.4692	502.7382			K	***766.3842***	383.6958	749.3577	375.1825	748.3737	374.6905	6
10	**1136.5227**	568.7650	1119.4962	560.2517	1118.5121	559.7597	D	***638.2893***	319.6483	621.2627	311.1350	620.2787	310.6430	5
11	1299.5860	650.2967	1282.5595	641.7834	1281.5755	641.2914	Y	***523.2623***	262.1348	506.2358	253.6215			4
12	**1413.6290**	707.3181	1396.6024	698.8048	1395.6184	698.3128	N	***360.1990***	180.6031	343.1724	172.0899			3
13	1484.6661	742.8367	1467.6395	734.3234	1466.6555	733.8314	A	***246.1561***	123.5817	229.1295	115.0684			2
14							R	175.1190	88.0631	158.0924	79.5498			1

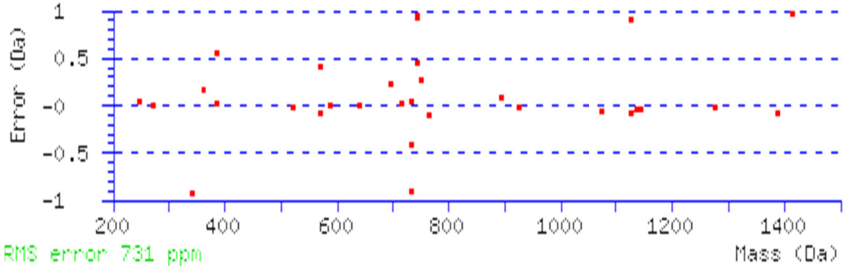

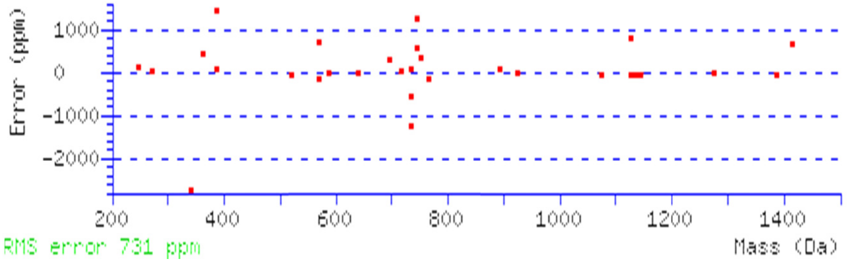

MS/MS Fragmentation of **GVNIMAFCKDYNAR**
Found in **AT1G32990.1** in ATH_Cplet_D, | Symbols: **PRPL11** | plastid ribosomal protein l11 | chr1:11955827-11957139 FORWARD LENGTH=222

Match to Query 3130: 1715.806332 from(572.942720,3+) intensity(551672.7500) scans(4281) rawscans(sn4281) rtinseconds(1545.4974) index(3424)

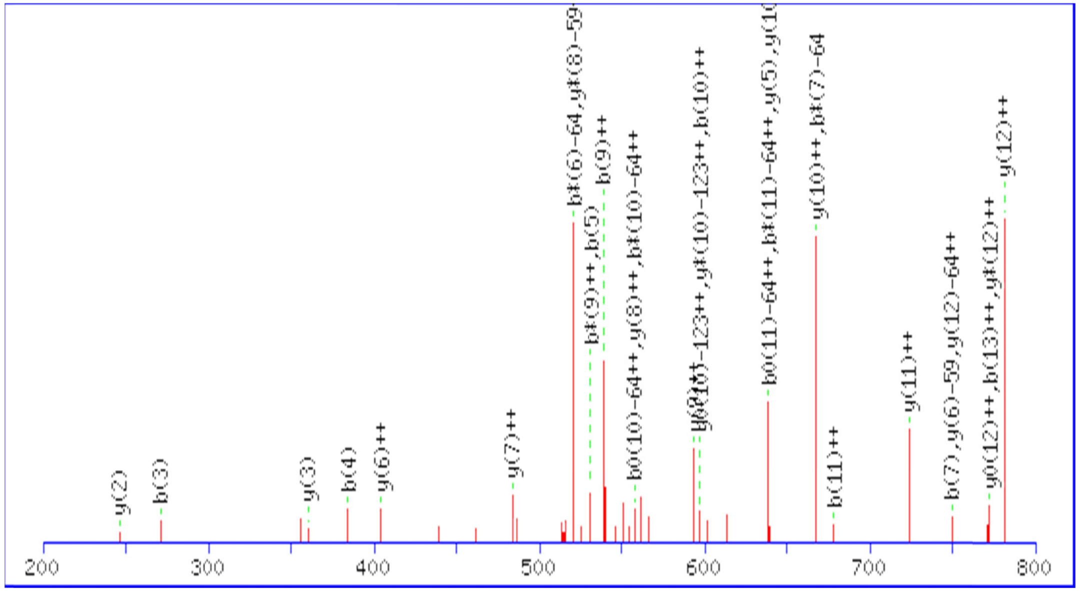

Monoisotopic mass of neutral peptide Mr(calc): 1715.8123
Fixed modifications: Carbamidomethyl (C) (apply to specified residues or termini only)
Variable modifications:
M5 : Oxidation (M), with neutral losses 0.0000(shown in table), 63.9983
K9 : Trimethyl (K), with neutral losses 0.0000(shown in table), 59.0735
Ions Score: 57 **Expect:** 0.00056
Matches : 33/322 fragment ions using 28 most intense peaks

#	b	b^{++}	b*	b^{*++}	b^{0}	b^{0++}	Seq.	y	y^{++}	y*	y^{*++}	y^{0}	y^{0++}	#
1	58.0287	29.5180					G							14
2	157.0972	79.0522					V	1659.7982	830.4027	1642.7716	821.8894	1641.7876	821.3974	13
3	**271.1401**	136.0737	254.1135	127.5604			N	1560.7297	780.8685	1543.7032	772.3552	1542.7192	771.8632	12
4	**384.2241**	192.6157	367.1976	184.1024			I	1446.6868	723.8470	1429.6603	715.3338	1428.6763	714.8418	11
5	**531.2595**	266.1334	514.2330	257.6201			M	1333.6028	667.3050	1316.5762	658.7917	1315.5922	658.2997	10
6	602.2967	301.6520	585.2701	293.1387			A	1186.5674	593.7873	1169.5408	585.2740	1168.5568	584.7820	9
7	**749.3651**	375.1862	732.3385	366.6729			F	1115.5302	558.2688	1098.5037	549.7555	1097.5197	549.2635	8
8	909.3957	455.2015	892.3692	446.6882			C	968.4618	484.7346	951.4353	476.2213	950.4513	475.7293	7
9	1079.5376	***540.2725***	1062.5111	531.7592			K	808.4312	404.7192	791.4046	396.2060	790.4206	395.7139	6
10	1194.5646	***597.7859***	1177.5380	589.2727	1176.5540	588.7806	D	**638.2893**	319.6483	621.2627	311.1350	620.2787	310.6430	5
11	1357.6279	***679.3176***	1340.6014	670.8043	1339.6173	670.3123	Y	523.2623	262.1348	506.2358	253.6215			4
12	1471.6708	736.3391	1454.6443	727.8258	1453.6603	727.3338	N	**360.1990**	180.6031	343.1724	172.0899			3
13	1542.7079	***771.8576***	1525.6814	763.3443	1524.6974	762.8523	A	**246.1561**	123.5817	229.1295	115.0684			2
14							R	175.1190	88.0631	158.0924	79.5498			1

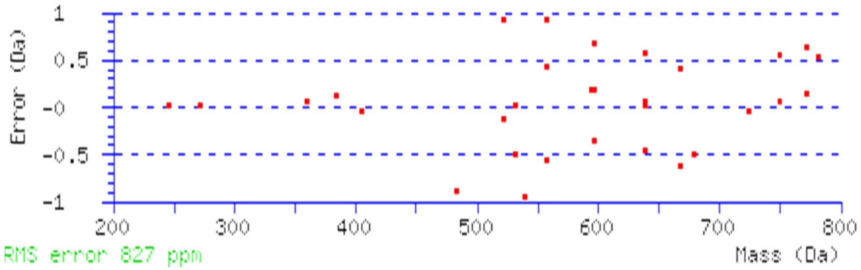

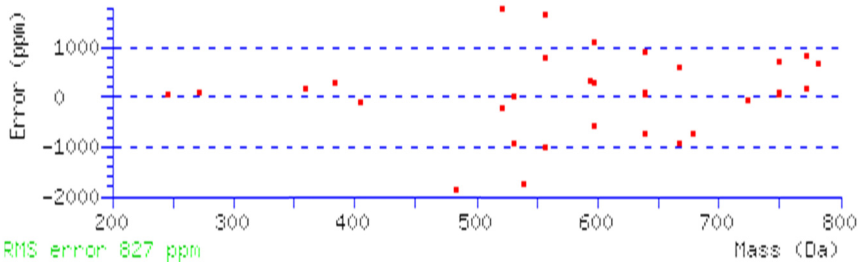

MS/MS Fragmentation of **FLSQPFFVAEVFTGSPGK**
Found in **ATCG00480.1** in ATH_Cplet_D, | Symbols: **ATPB**, PB | ATP synthase subunit beta | chrC:52660-54156 REVERSE LENGTH=498

Match to Query 7280: 1956.997808 from(979.506180,2+) intensity(229304.4100) scans(13629) rawscans(sn13629) rtinseconds(2907.929) index(11798)

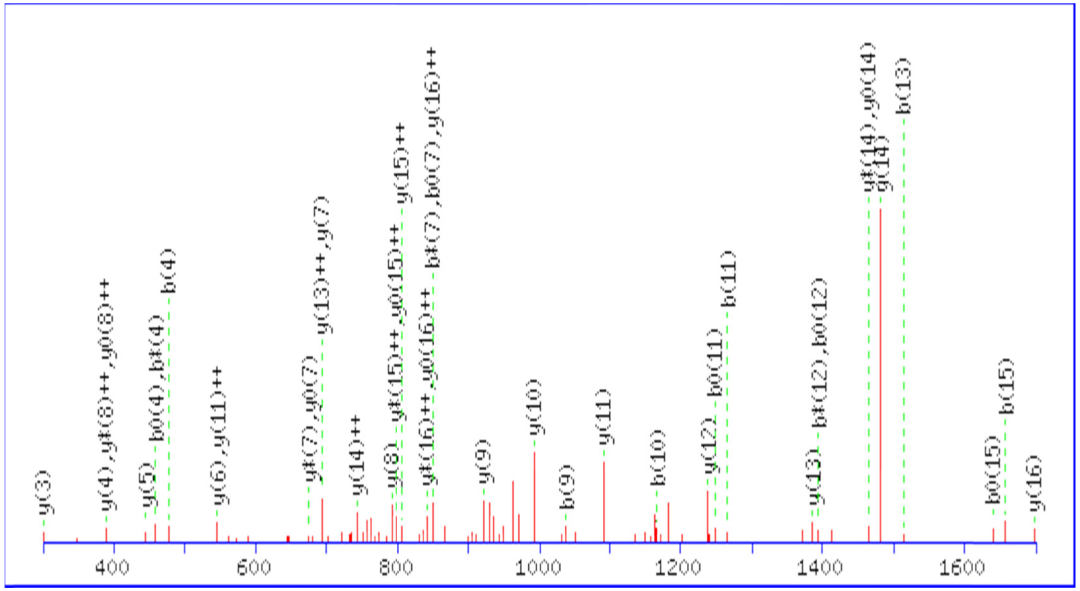

Monoisotopic mass of neutral peptide Mr(calc): 1956.9986
Fixed modifications: Carbamidomethyl (C) (apply to specified residues or termini only)
Ions Score: 113 **Expect:** 2.6e-09
Matches : 42/188 fragment ions using 40 most intense peaks

#	b	b^{++}	b*	b^{*++}	b^{0}	b^{0++}	Seq.	y	y^{++}	y*	y^{*++}	y^{0}	y^{0++}	#
1	148.0757	74.5415					F							18
2	261.1598	131.0835					L	1810.9374	905.9723	1793.9109	897.4591	1792.9268	896.9671	17
3	348.1918	174.5995			330.1812	165.5942	S	***1697.8534***	849.4303	1680.8268	840.9170	1679.8428	840.4250	16
4	**476.2504**	238.6288	459.2238	230.1155	458.2398	229.6235	Q	1610.8213	805.9143	1593.7948	797.4010	1592.8108	796.9090	15
5	573.3031	287.1552	556.2766	278.6419	555.2926	278.1499	P	***1482.7627***	741.8850	1465.7362	733.3717	1464.7522	732.8797	14
6	720.3715	360.6894	703.3450	352.1761	702.3610	351.6841	F	***1385.7100***	693.3586	1368.6834	684.8454	1367.6994	684.3533	13
7	867.4400	434.2236	850.4134	425.7103	849.4294	425.2183	F	***1238.6416***	619.8244	1221.6150	611.3111	1220.6310	610.8191	12
8	966.5084	483.7578	949.4818	475.2445	948.4978	474.7525	V	***1091.5732***	546.2902	1074.5466	537.7769	1073.5626	537.2849	11
9	**1037.5455**	519.2764	1020.5189	510.7631	1019.5349	510.2711	A	***992.5047***	496.7560	975.4782	488.2427	974.4942	487.7507	10
10	**1166.5881**	583.7977	1149.5615	575.2844	1148.5775	574.7924	E	***921.4676***	461.2375	904.4411	452.7242	903.4571	452.2322	9
11	**1265.6565**	633.3319	1248.6299	624.8186	1247.6459	624.3266	V	***792.4250***	396.7162	775.3985	388.2029	774.4145	387.7109	8
12	1412.7249	706.8661	1395.6984	698.3528	1394.7143	697.8608	F	***693.3566***	347.1819	676.3301	338.6687	675.3461	338.1767	7
13	**1513.7726**	757.3899	1496.7460	748.8767	1495.7620	748.3846	T	***546.2882***	273.6477	529.2617	265.1345	528.2776	264.6425	6
14	1570.7940	785.9007	1553.7675	777.3874	1552.7835	776.8954	G	***445.2405***	223.1239	428.2140	214.6106	427.2300	214.1186	5
15	**1657.8261**	829.4167	1640.7995	820.9034	1639.8155	820.4114	S	***388.2191***	194.6132	371.1925	186.0999	370.2085	185.6079	4
16	1754.8788	877.9431	1737.8523	869.4298	1736.8683	868.9378	P	***301.1870***	151.0972	284.1605	142.5839			3
17	1811.9003	906.4538	1794.8738	897.9405	1793.8897	897.4485	G	204.1343	102.5708	187.1077	94.0575			2
18							K	147.1128	74.0600	130.0863	65.5468			1

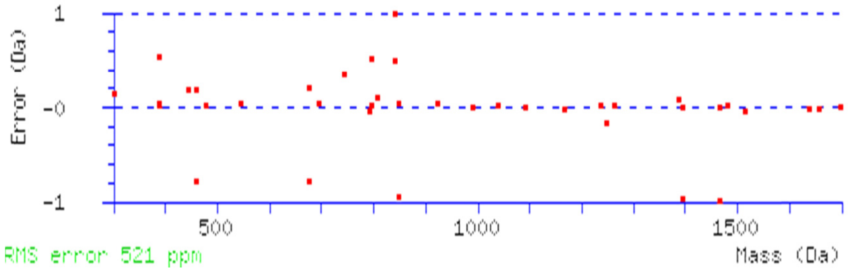

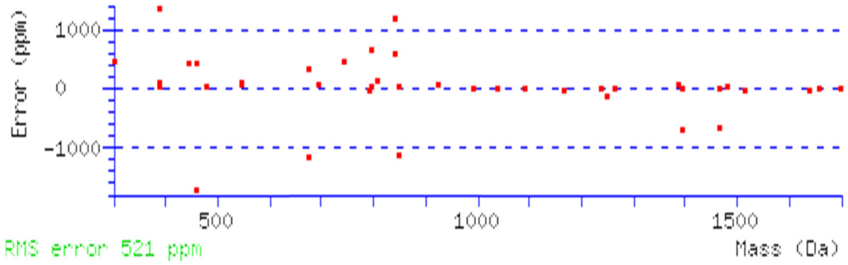

MS/MS Fragmentation of **FLSQPFFVAEVFTGSPGKYVGLAETIR**
Found in **ATCG00480.1** in ATH_Cplet_D, | Symbols: **ATPB**, PB | ATP synthase subunit beta | chrC:52660-54156 REVERSE LENGTH=498

Match to Query 11721: 2987.582682 from(996.868170,3+) intensity(9231552.0000) scans(12814) rawscans(sn12814) rtinseconds(2692.978) index(10946)

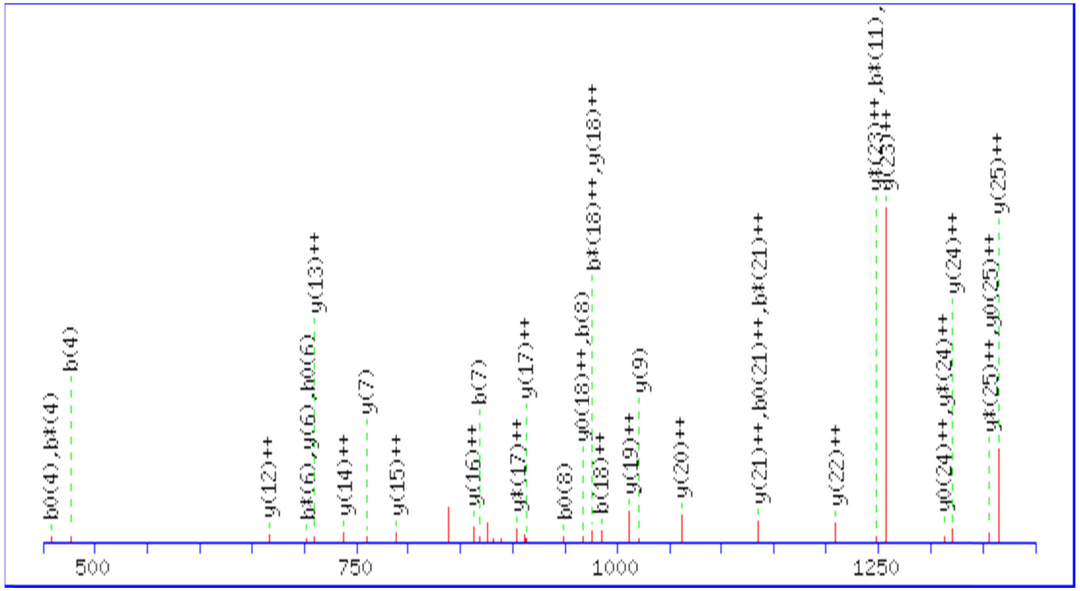

Monoisotopic mass of neutral peptide Mr(calc): 2987.5797
Fixed modifications: Carbamidomethyl (C) (apply to specified residues or termini only)
Variable modifications:
K18 : Dimethyl (K)
Ions Score: 122 **Expect:** 3.3e-10
Matches : 39/298 fragment ions using 30 most intense peaks

#	b	b^{++}	b^{*}	b^{*++}	b^{0}	b^{0++}	Seq.	y	y^{++}	y^{*}	y^{*++}	y^{0}	y^{0++}	#
1	148.0757	74.5415					F							27
2	261.1598	131.0835					L	2841.5185	1421.2629	2824.4920	1412.7496	2823.5080	1412.2576	26
3	348.1918	174.5995			330.1812	165.5942	S	2728.4345	1364.7209	2711.4079	1356.2076	2710.4239	1355.7156	25
4	**476.2504**	238.6288	459.2238	230.1155	458.2398	229.6235	Q	2641.4025	1321.2049	2624.3759	1312.6916	2623.3919	1312.1996	24
5	573.3031	287.1552	556.2766	278.6419	555.2926	278.1499	P	2513.3439	1257.1756	2496.3173	1248.6623	2495.3333	1248.1703	23
6	720.3715	360.6894	703.3450	352.1761	702.3610	351.6841	F	2416.2911	1208.6492	2399.2646	1200.1359	2398.2805	1199.6439	22
7	**867.4400**	434.2236	850.4134	425.7103	849.4294	425.2183	F	2269.2227	1135.1150	2252.1961	1126.6017	2251.2121	1126.1097	21
8	**966.5084**	483.7578	949.4818	475.2445	948.4978	474.7525	V	2122.1543	1061.5808	2105.1277	1053.0675	2104.1437	1052.5755	20
9	1037.5455	519.2764	1020.5189	510.7631	1019.5349	510.2711	A	2023.0859	1012.0466	2006.0593	1003.5333	2005.0753	1003.0413	19
10	1166.5881	583.7977	1149.5615	575.2844	1148.5775	574.7924	E	1952.0488	976.5280	1935.0222	968.0147	1934.0382	967.5227	18
11	1265.6565	633.3319	1248.6299	624.8186	1247.6459	624.3266	V	1823.0062	912.0067	1805.9796	903.4934	1804.9956	903.0014	17
12	1412.7249	706.8661	1395.6984	698.3528	1394.7143	697.8608	F	1723.9377	862.4725	1706.9112	853.9592	1705.9272	853.4672	16
13	1513.7726	757.3899	1496.7460	748.8767	1495.7620	748.3846	T	1576.8693	788.9383	1559.8428	780.4250	1558.8588	779.9330	15
14	1570.7940	785.9007	1553.7675	777.3874	1552.7835	776.8954	G	1475.8217	738.4145	1458.7951	729.9012	1457.8111	729.4092	14
15	1657.8261	829.4167	1640.7995	820.9034	1639.8155	820.4114	S	1418.8002	709.9037	1401.7736	701.3905	1400.7896	700.8985	13
16	1754.8788	877.9431	1737.8523	869.4298	1736.8683	868.9378	P	1331.7682	666.3877	1314.7416	657.8744	1313.7576	657.3824	12
17	1811.9003	906.4538	1794.8738	897.9405	1793.8897	897.4485	G	1234.7154	617.8613	1217.6889	609.3481	1216.7048	608.8561	11
18	1968.0266	984.5169	1951.0000	976.0036	1950.0160	975.5116	K	1177.6939	589.3506	1160.6674	580.8373	1159.6834	580.3453	10
19	2131.0899	1066.0486	2114.0633	1057.5353	2113.0793	1057.0433	Y	**1021.5677**	511.2875	1004.5411	502.7742	1003.5571	502.2822	9
20	2230.1583	1115.5828	2213.1318	1107.0695	2212.1477	1106.5775	V	858.5043	429.7558	841.4778	421.2425	840.4938	420.7505	8
21	2287.1798	1144.0935	2270.1532	1135.5802	2269.1692	1135.0882	G	**759.4359**	380.2216	742.4094	371.7083	741.4254	371.2163	7
22	2400.2638	1200.6356	2383.2373	1192.1223	2382.2533	1191.6303	L	**702.4145**	351.7109	685.3879	343.1976	684.4039	342.7056	6
23	2471.3009	1236.1541	2454.2744	1227.6408	2453.2904	1227.1488	A	589.3304	295.1688	572.3039	286.6556	571.3198	286.1636	5
24	2600.3435	1300.6754	2583.3170	1292.1621	2582.3330	1291.6701	E	518.2933	259.6503	501.2667	251.1370	500.2827	250.6450	4
25	2701.3912	1351.1992	2684.3647	1342.6860	2683.3807	1342.1940	T	389.2507	195.1290	372.2241	186.6157	371.2401	186.1237	3
26	2814.4753	1407.7413	2797.4487	1399.2280	2796.4647	1398.7360	I	288.2030	144.6051	271.1765	136.0919			2
27							R	175.1190	88.0631	158.0924	79.5498			1

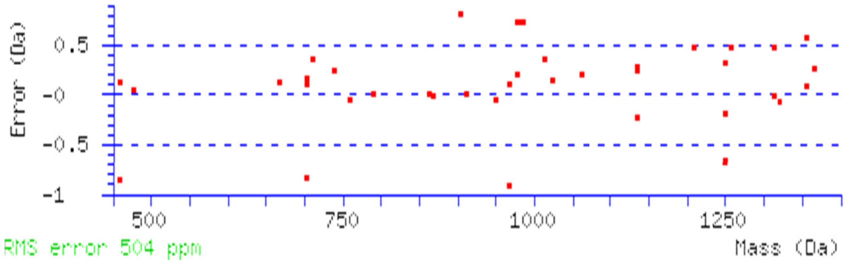

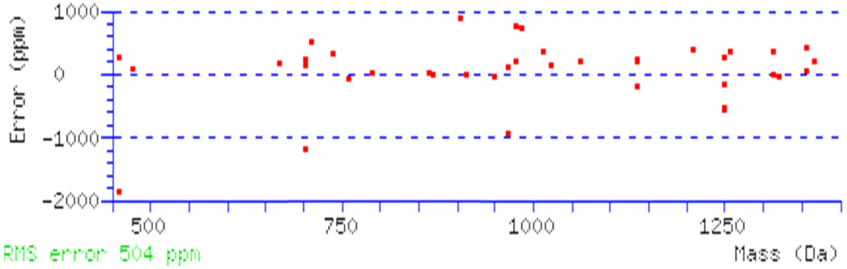

Supplement: Figure S4 — LC-MS/MS fragmentation spectra of recombinant PRPL11 and ATP-B methylated in vitro by chloroplast stroma. (PDF) [file pone.0095512.s004.pdf]
