# Supplementary material for: Uncovering the Protein Lysine and Arginine Methylation Network in Arabidopsis Chloroplasts
Source: PLoS One. 2014 Apr 18;9(4):e95512. doi: 10.1371/journal.pone.0095512 (PMC3991674; doi:10.1371/journal.pone.0095512)
Supplement: Table S1 — Oligonucleotides used in this study. (PDF) [file pone.0095512.s005.pdf]

Table S1: Oligonucleotides used in this study.

Name	Sequence (5' → 3')
ATPB-pETNdeI	CATATGAGAACAAATCCTACTACTT
ATPB-pETEcoRI	GAATTCTCATTTCTTCAATTTACTCTC
ATPB-K447A	GTATTTACAGGTTCTCCGGGAGCATATGTTGGTCTAGCGGAAAC
ATPB-K447A-anti	GTTTCCGCTAGACCAACATATGCTCCCGGAGAACCTGTAAATAC
ATPBMT-pETNdeI	CATATGGCGTCTCTGCCGTCACGG
ATPBMT-pETXhoI	CTCGAGTGCAGCAGTTGGTGTCTC
GAPA1-pETNdeI	CATATGGCCAAGCTTAAGGTGGC
GAPA1-pETBamHI	GGATCCTCACTTCCAGTTGTTGGC
PrmA-pETNdeI	CATATGTCTTTCGCTGCACCTTAC
PrmA-pETXhoI	CTCGAGGTTATCAATAAACTCCTCTTTCTTGG
PRPL11-pETNdeI	CATATGGCTCCACCTAAACCC
PRPL11-pETXhoI	CTCGAGCAATAAAACTGCTTTCTTTTTGGG
